# Nasal Mucosa‐Derived Extracellular Vesicles as a Systemic Antiaging Intervention

**DOI:** 10.1002/advs.202511372

**Published:** 2025-11-21

**Authors:** Wentao Shi, Lu Bian, Mengyuan Yu, Jun Wang, Mingzhu Gao, Liping Shen, Xiao Zhang, Zhiliang Huang, Hong Tang, Long Lv, Yunduan Que, Zengli Miao, Xuechao Wu, Qing Wang, Xiaojie Lu

**Affiliations:** ^1^ Department of Neurosurgery Jiangnan University Medical Center Wuxi No. 2 People's Hospital Wuxi Jiangsu Province 214122 China; ^2^ Department of Central Laboratory Jiangsu University Affiliated Gaochun Hospital Jiangsu University Nanjing Jiangsu Province 211300 China; ^3^ Wuxi Neurosurgical Institute Wuxi Jiangsu Province 214122 China; ^4^ Department of Neuroscience Center Wuxi School of Medicine Jiangnan University Wuxi Jiangsu Province 214122 China

**Keywords:** anti‐aging, EVs, nasal mucosa

## Abstract

Aging impairs tissue function and regenerative capacity across multiple organs. This study demonstrates that extracellular vesicles derived from human nasal mucosa (nmEVs) exert systemic antiaging effects in aged mice. Treatment with nmEVs improves cognitive performance and alters hippocampal aging signatures related to synaptic signaling and the regulation of neuroplasticity. In parallel, transcriptomic analysis of five major aging‐sensitive organs reveals that nmEVs broadly ameliorate age‐associated transcriptional changes, notably by restoring circadian rhythmicity and suppressing cellular senescence‐related pathways. At the cellular level, nmEVs alleviate senescence phenotypes in aged human bone marrow mesenchymal stem cells, restore proliferation and osteogenic capacity, and reactivate core clock gene expression. These effects are accompanied by modulation of the p53 pathway, suggesting its involvement in nmEV‐mediated rejuvenation. Importantly, lacking the need for cell isolation and ex vivo expansion, nmEVs offer a practical, age‐independent source of extracellular vesicles with high clinical accessibility. Together, these findings support the translational potential of nmEVs as a multifaceted therapeutic candidate for systemic aging intervention.

## Graphical abstract

This study shows that nasal mucosa‐derived extracellular vesicles (nmEVs) exert systemic anti‐ageing effects in mice by restoring circadian rhythm, suppressing cellular senescence, and improving cognitive function. In aged human bone marrow mesenchymal stem cells, nmEVs reverse senescence‐associated phenotypes and reactivate core clock gene expression.

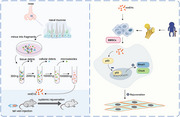

## 1 Introduction

Aging is characterized by a progressive decline in physiological integrity, leading to impaired tissue homeostasis, reduced regenerative capacity and increased vulnerability to disease.^[^ ^1^ ^]^ Hallmarks of aging include genomic instability, telomere attrition, epigenetic alterations, deregulated nutrient sensing, mitochondrial dysfunction, cellular senescence, stem cell exhaustion and altered intercellular communication.^[^ ^2^ ^]^ To counteract these changes, a range of rejuvenation strategies have been proposed, including metabolic modulation, clearance of senescent cells and the use of extracellular vesicles (EVs) derived from regenerative tissues.^[^ ^3^ ^]^

EVs are nanoscale membrane‐bound particles released by virtually all cell types.^[^ ^4^ ^]^ They serve as key mediators of intercellular communication by transferring proteins, small RNAs and DNA between cells.^[^ ^5^ ^]^ The use of EVs, particularly those derived from stem cells, which have shown promising rejuvenating effects, in aging research has attracted increasing interest.^[^ ^6^, ^7^ ^]^ However, EVs derived from specialized tissues with intrinsic regenerative capacity remain largely unexplored. Identifying such tissues and harnessing their EVs may offer a novel and potent antiaging strategy.

**Scheme 1:**
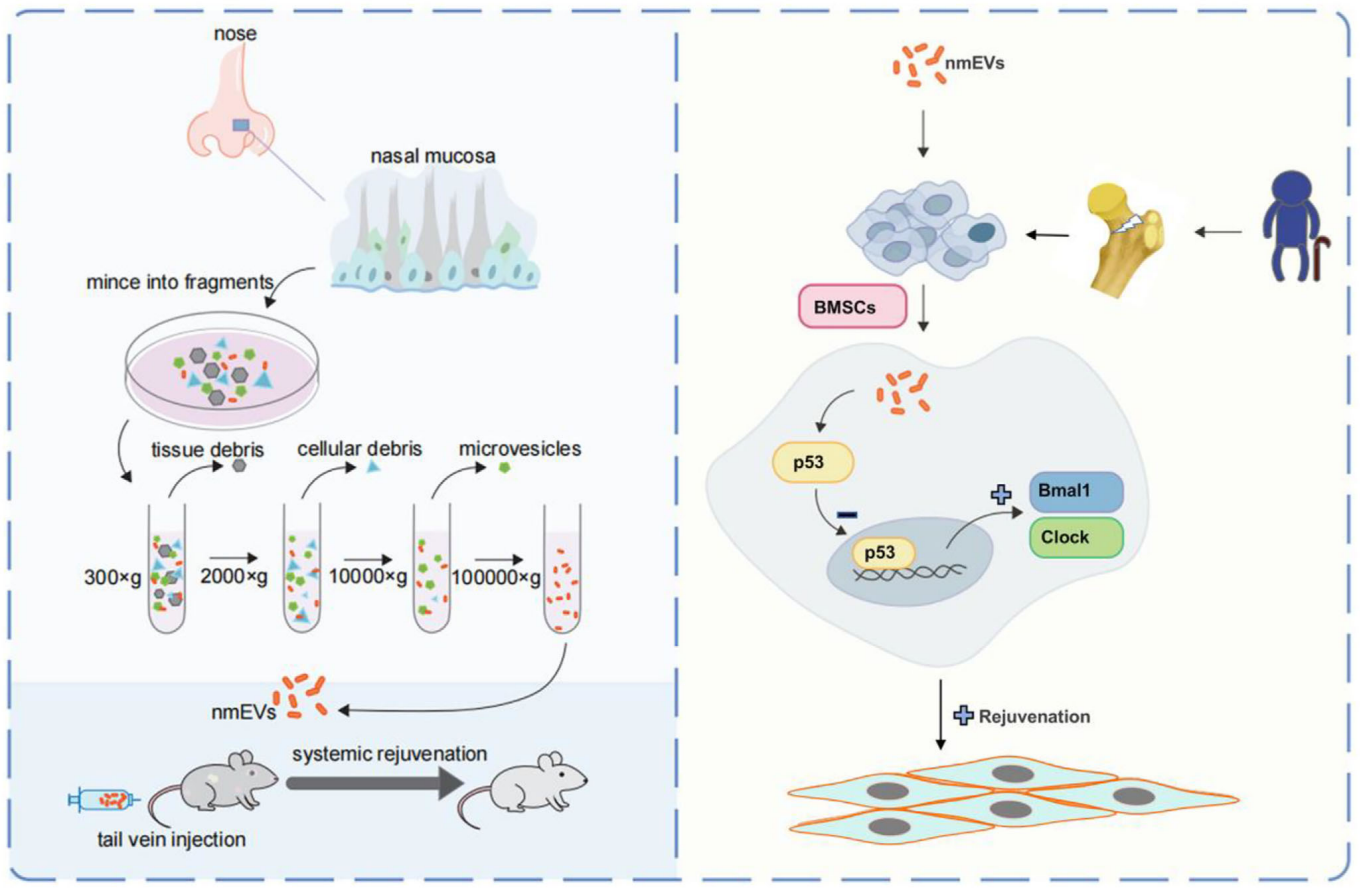
Schematic illustration of the ability of nmEVs to rejuvenate multiple organs and reverse cellular aging via systemic administration.

Endoscopic transsphenoidal surgery for pituitary adenoma often requires harvesting the pedicled nasal mucosa for skull base reconstruction.^[^ ^8^ ^]^ Unexpectedly, even after excising tissue segments up to 2 × 2 cm, the nasal mucosa regenerates rapidly and seamlessly, with no apparent scarring. This regenerative capacity is consistently observed even in elderly individuals and far exceeds that of most other tissues. Such a phenomenon suggests that the nasal mucosa retains remarkable youthful, anti‐senescence properties, irrespective of age. Thus, we hypothesized that EVs derived from the nasal mucosa possess intrinsic antiaging properties. To test this hypothesis, we isolated EVs from human nasal mucosal tissue and administered them to aged mice via tail vein injection. Comprehensive in vivo analyses were conducted to assess their systemic effects, with a particular focus on the brain, liver and lungs, which are organs critically affected by aging. In parallel, we isolated and cultured bone marrow mesenchymal stem cells (BMSCs) from elderly donors and treated them with nasal mucosa‐derived EVs (nmEVs) to evaluate their antiaging effects at the cellular level. A schematic overview of the experimental design, including EV isolation, systemic and cellular treatment paradigms, and key outcomes, is illustrated in **Scheme** **1**, which summarizes the multilayered strategy used to evaluate the rejuvenating potential of nmEVs.

Our key findings are threefold: First, nmEVs improved cognitive performance in aged mice; second, they reduced aging‐associated molecular and histological markers across multiple organs; and third, both in vivo and in vitro evidence suggested that these effects were mediated, at least in part, by modulation of the p53 signaling pathway. Taken together, these findings revealed that nmEVs exert systemic antiaging effects in aged organisms at both the tissue and cellular levels. Their ability to increase cognitive function, attenuate organ‐specific aging markers and modulate the p53 signaling pathway highlights their therapeutic potential. Given the remarkable regenerative capacity and accessibility of nasal mucosa, its nmEVs represent a promising, tissue‐derived candidate for future antiaging interventions.

## 2 Results

### 2.1 Extracellular Vesicle Isolation and Characterization

**Figure 1:**
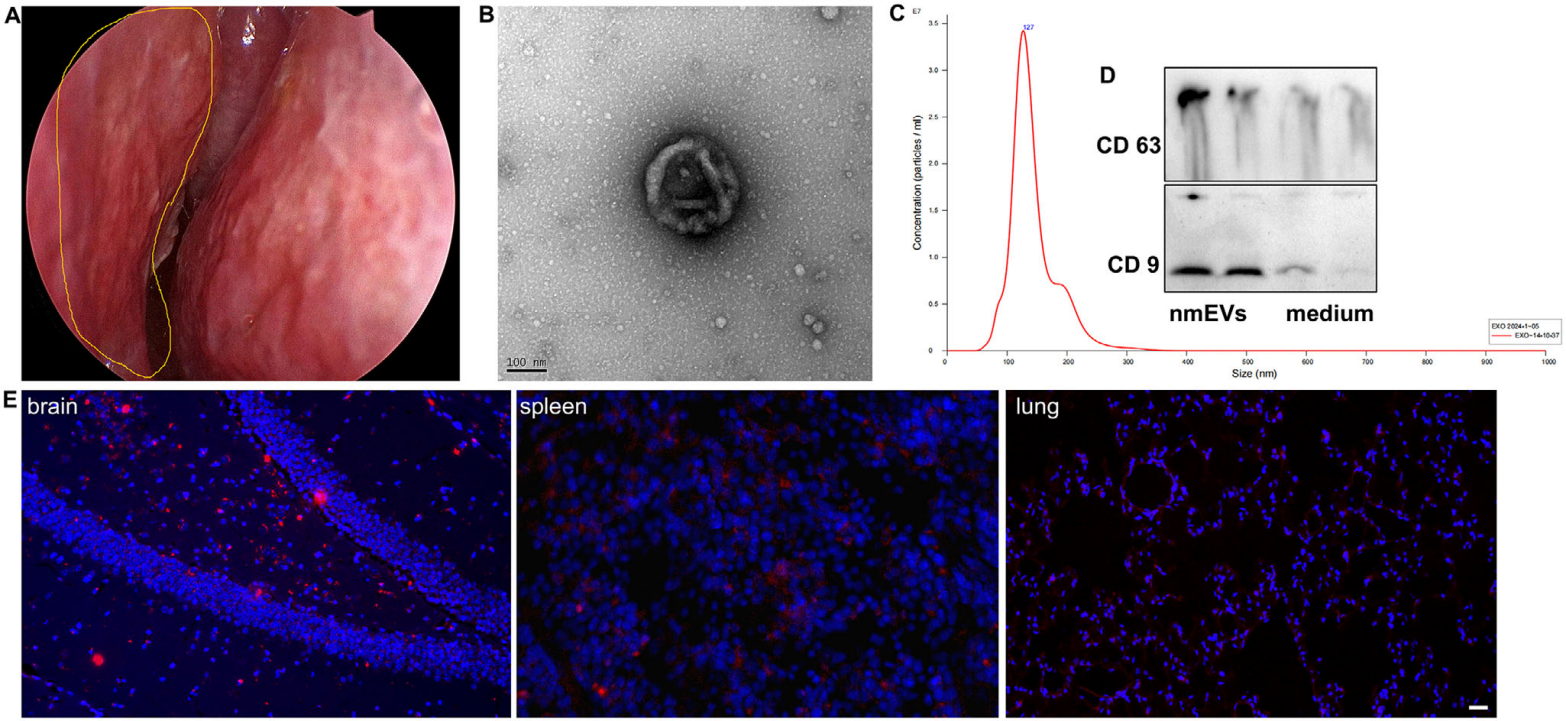
Characterization and in vivo biodistribution of nasal mucosa‐derived extracellular vesicles (nmEVs). A) Endoscopic image showing the nasal mucosa collection site. B) TEM image displaying spherical nmEVs with a typical morphology. C) NTA illustrating the size distribution of nmEVs. D) Western blot confirming the expression of CD63 and CD9 in nmEVs, with medium used as a negative control. E) PKH26‐labeled nmEVs were intravenously administered to aged mice. Representative fluorescence images show distribution in the brain, spleen, and lungs. Bar = 20 µm.

To investigate the regenerative and antiaging potential of nmEVs, we first isolated EVs from conditioned media via differential ultracentrifugation in accordance with the Minimal Information for Studies of Extracellular Vesicles (MISEV) 2018 guidelines. Human nasal mucosa was harvested endoscopically from the respiratory region of the superior turbinate, and representative intraoperative images were captured to illustrate the anatomical site and collection procedure (**Figure** **1**A).

Transmission electron microscopy (TEM) revealed spherical vesicles ranging from 50 to 300 nm in diameter that exhibited classical EV morphology (Figure 1B). Nanoparticle tracking analysis (NTA) revealed a particle size distribution consistent with that of EVs, with a mean diameter of 169 nm and a modal size of 176 nm (Figure 1C). Immunoblot analysis confirmed the presence of the cytosolic EV markers CD63 and CD9 in the isolated samples,^[^ ^9^ ^]^ whereas the medium derived from trace exosome‐depleted fetal bovine serum served as a control for potential plasma protein contamination (Figure 1D).

To assess biodistribution in vivo, EVs were labeled with the lipophilic dye PKH26 and intravenously administered to aged mice. Fluorescent signals were subsequently detected in the brain, spleen and lungs, indicating efficient delivery and tissue uptake of the nasally derived EVs in organs commonly affected by aging (Figure 1E). These findings establish a foundation for evaluating the functional relevance of nmEVs in aging‐associated physiological decline.

### 2.2 Nasal Mucosa‐Derived EVs Improve the Health Span of Aged Mice

Aging is typically associated with a progressive decline in physical capacity and an increased risk of frailty.^[^ ^10^, ^11^ ^]^ To investigate whether nmEVs could improve overall physical performance in aged organisms, we conducted a battery of physiological tests in aged mice (20 months old), with younger mice (10 months old) used as a reference group and PBS‐treated aged mice used as parallel controls. Prior to treatment, baseline measurements of grip strength, fatigue resistance, and motor coordination were obtained across all groups.

**Figure 2:**
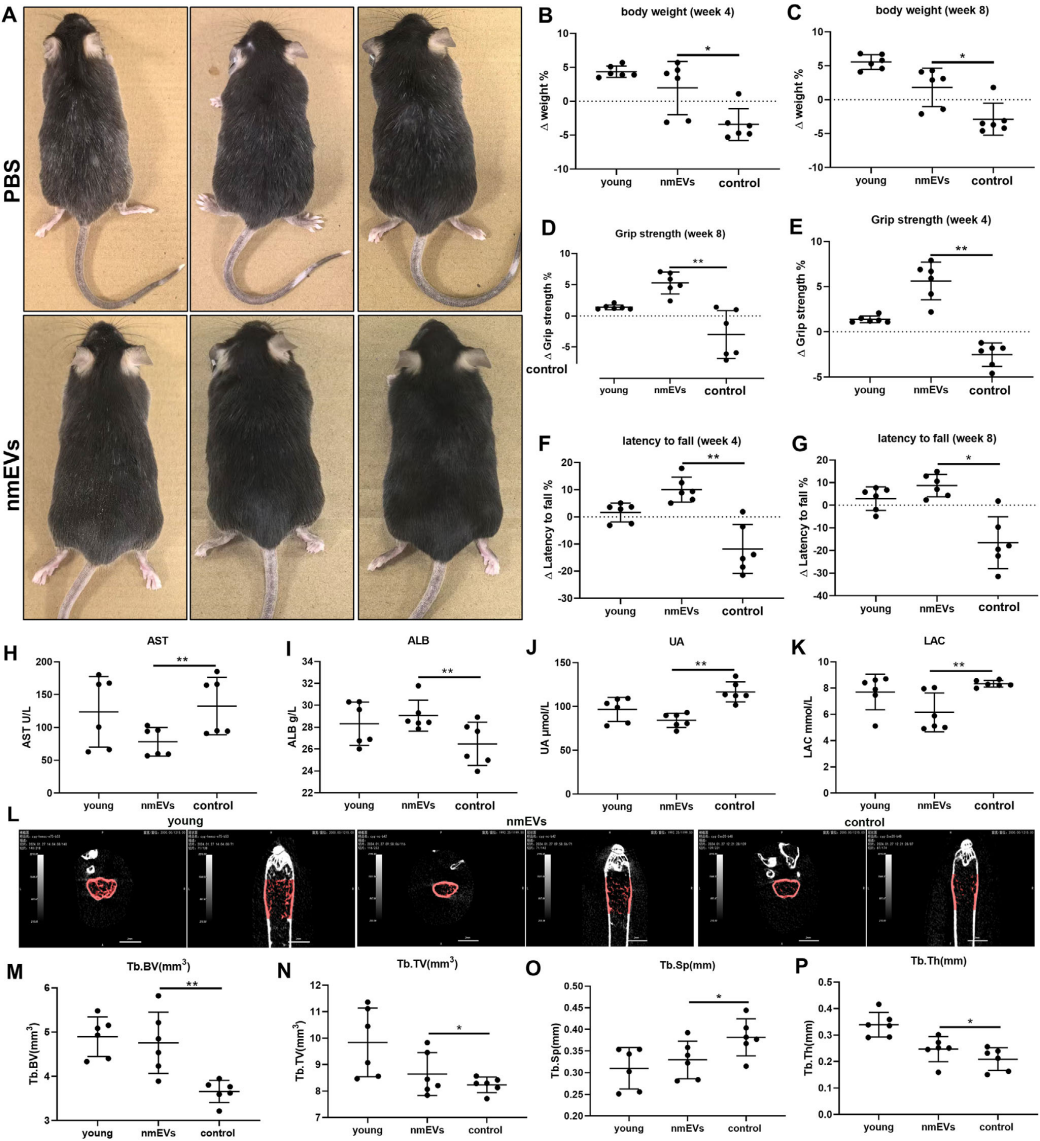
nmEVs improve physical function and attenuate skeletal aging in aged mice. A) Representative images of mice before and 8 weeks after treatment with nmEVs or PBS. B,C) Body weight remained stable across groups, and the data are shown as the change in percentage from baseline before treatment. D,E) Grip strength was significantly increased in nmEV‐treated mice; data are shown as the change in percentage from baseline before treatment. F,G) Rotarod test showing increased motor coordination in the nmEV group; data are shown as the change in percentage from baseline before treatment. H–K) Plasma biochemistry analysis revealed reduced AST, albumin, uric acid, and lactate levels. L) Representative 3D micro‐CT reconstructions of the tibiae of nmEV‐treated mice showing the preserved trabecular bone structure. M–P) Quantitative micro‐CT analysis demonstrating improved Tb.BV, Tb.TV, Tb.BV/TV, and Tb.BS/TV in the nmEV group. Data are presented as mean ± SD (n = 6 biological replicates per group). Statistical significance was determined using one‐way ANOVA followed by Tukey's post‐hoc test for multiple group comparisons, or unpaired two‐tailed *t*‐test for pairwise comparisons, as appropriate. *p < 0.05, **p < 0.01, ***p < 0.001.

Aged mice then received intravenous injections of either nmEVs or PBS twice weekly for a total of 16 doses over 2 months. Throughout the treatment course, physical performance was assessed biweekly. No significant toxicity or adverse effects were observed in any group; all the animals were in good general health and had normal growth. Notably, the fur quality of nmEV‐treated mice clearly improved, with denser, darker, and glossier dorsal hair, in contrast to the sparse and grayish fur of PBS‐treated controls (**Figure** **2**A).

Body weight remained stable across all groups, with no significant differences before or after treatment (Figure 2B,C). However, by the end of the treatment period, nmEV‐treated aged mice demonstrated significant improvements in grip strength (Figure 2D,E) and rotarod performance for motor coordination (Figure 2F,G) compared with PBS‐treated control mice.

To evaluate systemic physiological status, plasma biochemistry analysis was performed at the study endpoint. Compared with control mice, nmEV‐treated mice presented lower levels of aspartate aminotransferase (Figure 2H), albumin (Figure 2I), uric acid (Figure 2J), and blood lactate (Figure 2K), which are markers often associated with metabolic stress and tissue injury. Other biochemical parameters, including kidney and electrolyte markers, remained within normal ranges and showed no significant group differences (Table S1, Supporting Information).

### 2.3 nmEVs Alleviate Age‐Associated Bone Loss

Bone is a dynamic organ that undergoes continuous remodeling to maintain structural integrity and mineral homeostasis.^[^ ^12^ ^]^ However, aging disrupts this balance by tipping the scale toward bone resorption, leading to progressive bone loss, microarchitectural deterioration, and increased fracture risk, collectively termed age‐related osteoporosis.^[^ ^13^ ^]^ Cellular senescence and chronic low‐grade inflammation within the bone microenvironment are increasingly recognized as key drivers of this process.^[^ ^14^ ^]^ Given the previously demonstrated anti‐senescence and anti‐inflammatory effects of nmEVs in multiple tissues, we investigated whether nmEVs could alleviate skeletal aging and preserve bone quality in aged mice.

To evaluate the protective effects of nmEVs on age‐related bone deterioration, we performed microcomputed tomography (micro‐CT) analysis of the proximal tibiae of three groups of mice: aged controls, aged mice treated with nmEVs, and young controls. Representative 3D micro‐CT reconstructions of the proximal tibia from each group visually highlight the protective effects of nmEVs on trabecular bone structure (Figure 2L). Aged control mice exhibited sparse and disconnected trabeculae, with evident thinning and large intertrabecular spaces, which are hallmarks of age‐related bone loss. In contrast, mice treated with nmEVs displayed a denser, more interconnected trabecular network, with improved bone volume and microarchitectural integrity, closely resembling those observed in young mice. Afterward, a range of structural and mineral parameters were quantified to assess trabecular bone quality.

To evaluate the effects of nmEVs on bone microstructure in aged mice, we performed micro‐CT analysis of the proximal tibiae and quantified six representative structural parameters. Trabecular bone volume (Tb.BV) reflects the total amount of mineralized bone present within the region of interest. Compared with PBS‐treated aged controls, nmEV‐treated mice exhibited significantly increased Tb.BV (Figure 2M), indicating a substantial reversal of trabecular bone loss. The total volume (Tb.TV), which reflects the entire volume of the analyzed region, was not significantly different between the groups (Figure 2N), suggesting that the observed improvements were not due to gross anatomical changes but rather to changes in internal microarchitecture. The bone volume fraction (Tb.BV/TV), the ratio of trabecular bone volume to total tissue volume, was also significantly elevated in nmEV‐treated mice (Figure 2O), reflecting the improved density and occupancy of trabecular bone within the marrow space. Bone surface density (Tb.BS/TV), an indicator of trabecular surface area per unit volume and a proxy for remodeling potential, was significantly greater in the nmEV group than in the aged control group (Figure 2P), suggesting increased structural complexity and surface availability for osteoblast and osteoclast activity.

Collectively, these findings demonstrate that nmEV administration significantly attenuates key features of skeletal aging, improving bone volume, microarchitecture, and mineral density. These results provide strong evidence for the therapeutic potential of nmEVs in mitigating osteoporosis‐like changes associated with aging.

### 2.4 Single‐Nucleus RNA‐seq Reveals Cell Type‐Specific Responses to nmEVs in Hippocampal Tissue

**Figure 3:**
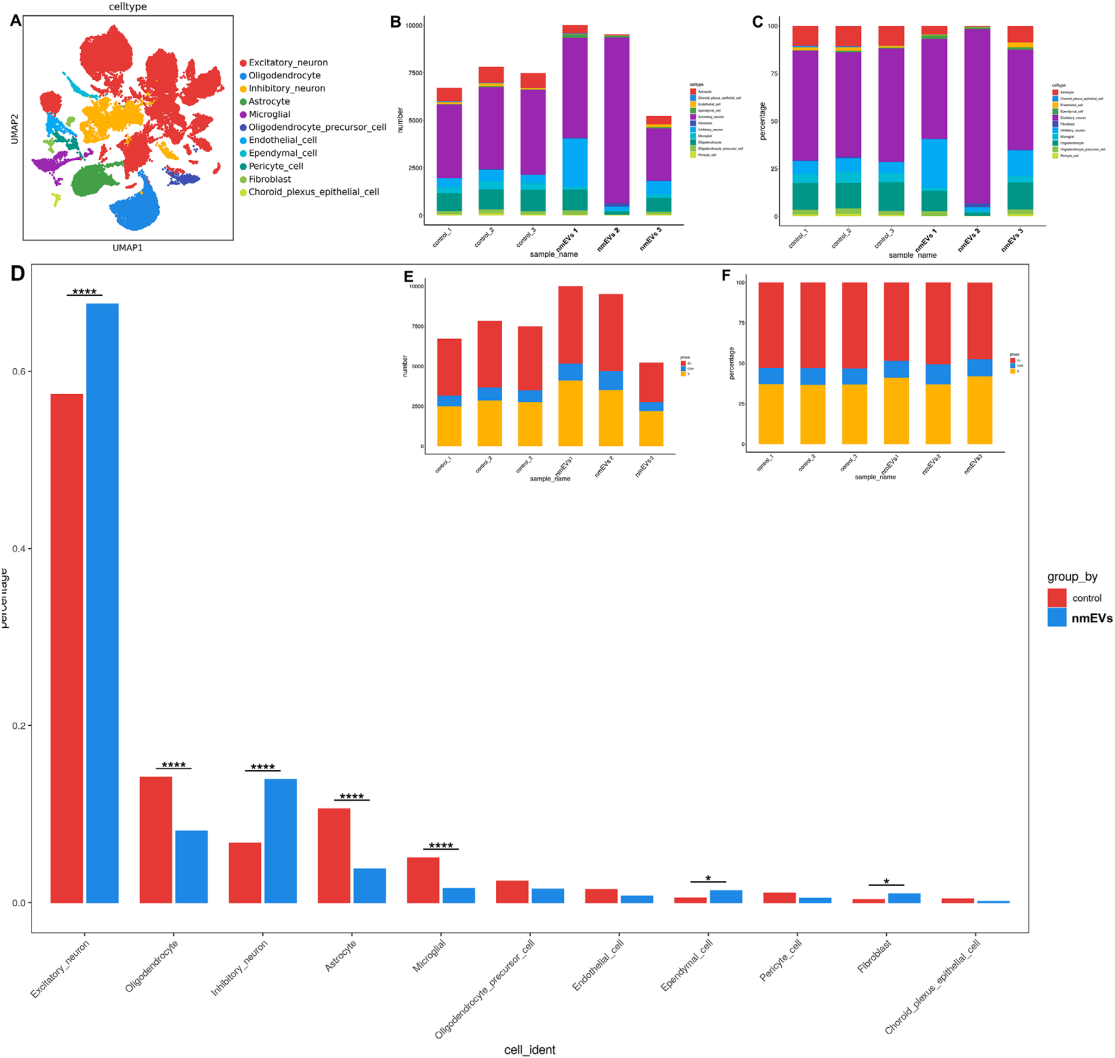
snRNA‐seq reveals neuro–glial remodeling by nmEVs in the hippocampus of aged animals. A) UMAP visualization of hippocampal cell populations across groups. B,C) Pie charts showing the relative cell type composition in the aged versus nmEV‐treated groups. D) Quantification of cell type proportions indicating increased numbers of neurons and reduced numbers of astrocytes/oligodendrocytes following nmEV treatment. E,F) Cell cycle analysis indicating increased proliferative activity in the nmEV group. Data are presented as the mean ± SEM from n = 3 biological replicates per group. Statistical significance was determined with a paired two‐tailed *t* test. *p < 0.05; **p < 0.01.

To explore age‐associated changes in hippocampal cell populations and assess the potential modulatory effects of nmEVs, we performed single‐nucleus RNA sequencing (snRNA‐seq) on hippocampal tissues from aged control mice and aged mice treated with nmEVs for 8 weeks. This approach enabled high‐resolution profiling of cellular heterogeneity and potential population shifts in response to intervention. Following stringent quality control and dimensionality reduction, the captured nuclei were clustered and classified into ten major cell types on the basis of canonical marker gene expression. These include excitatory neurons, inhibitory neurons, astrocytes, oligodendrocytes, oligodendrocyte precursor cells, ependymal cells, endothelial cells, fibroblasts, choroid plexus epithelial cells, and microglia. UMAP visualization of the cell populations is shown in **Figure** **3**A, and key marker gene expression profiles are summarized in Table S2, Supporting Information. To confirm the effective removal of batch effects and ensure an equitable contribution from each biological replicate, we visualized the integrated dataset in a low‐dimensional space with individual samples highlighted. As shown in Figure S1, Supporting Information, nuclei derived from different animals were evenly distributed across the UMAP embedding, without the formation of sample‐specific clusters.

In the control group, the cellular composition across these ten cell types remained relatively balanced, with no single population overwhelmingly dominant (Figure 3B). In contrast, the nmEV‐treated group exhibited notable shifts in cell type distribution, with certain neuronal subpopulations markedly expanded and glial components proportionally reduced (Figure 3C). Specifically, the number of excitatory and inhibitory neurons significantly increased in the nmEV‐treated group (67.6% versus 57.4%, *p* < 0.01; and 9.2% versus 6.7%, *p* < 0.01, respectively), accompanied by a marked reduction in the number of astrocytes and oligodendrocytes (10.6% versus 3.8%, *p* < 0.01; and 5.7% versus 2.4%, *p* < 0.01, respectively) (Figure 3D). To assess changes in proliferation, we analyzed the distribution of cell cycle phases across treatment groups. We observed a marked decrease in cells in the G1 phase and an increase in cells in both the S phase and the G2/M phase in the nmEV group, which indicated that nmEVs promote cell cycle progression and support regeneration (Figure 3E,F).

These observations suggest that aging disrupts hippocampal cell‐type equilibrium by promoting neuronal dominance and depleting supportive glial cells. Importantly, systemic administration of nmEVs appears to preserve a more balanced neuro–glial composition in the aged hippocampus.

### 2.5 Identification of Differentially Regulated Subclusters in the Aging Hippocampus

**Figure 4:**
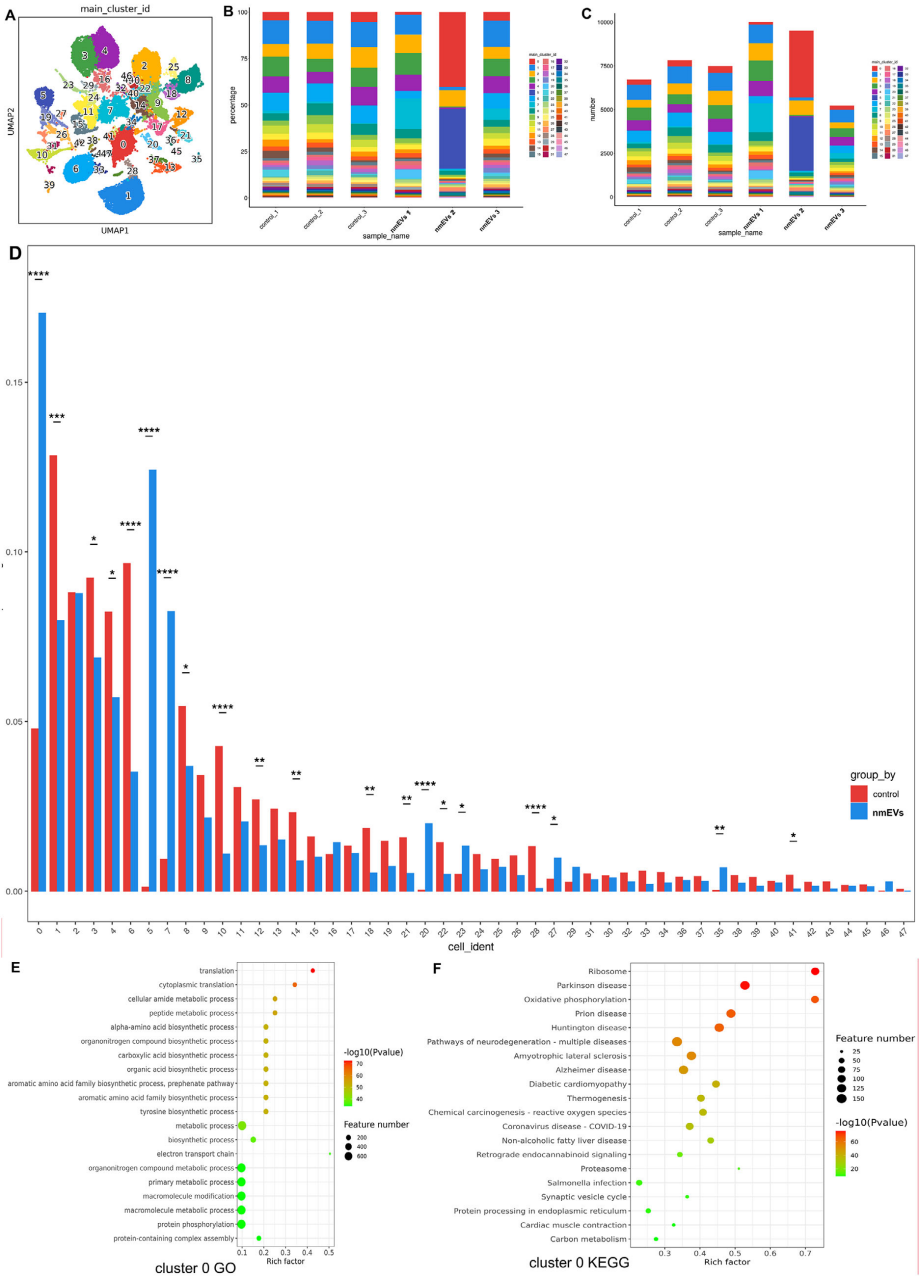
nmEVs selectively remodel transcriptionally distinct hippocampal subclusters. A) UMAP projection of 48 transcriptional clusters from hippocampal nuclei. B,C) Comparison of relative and absolute cluster sizes across groups. D) Five subclusters (Clusters 0, 6, 7, 10, and 20) were significantly altered by nmEVs, affecting excitatory/inhibitory neurons and astrocytes. E,F) Gene Ontology (GO) and Kyoto Encyclopedia of Genes and Genomes (KEGG) enrichment for Cluster 0 (excitatory neurons) highlight neuroplasticity, energy metabolism, and the oxidative stress response. Data are presented as the mean ± SEM from n = 3 biological replicates per group. Statistical significance was determined with a paired two‐tailed *t* test. *p < 0.05; **p < 0.01.

While the above analysis revealed significant differences in the overall proportions of several major cell types, each of these populations comprises multiple transcriptionally distinct subpopulations. To resolve this underlying heterogeneity, we returned to the original unsupervised clustering results, in which all the hippocampal nuclei were partitioned into 48 transcriptional clusters (**Figure** **4**A), and the key marker gene expression profiles are summarized in Table S3, Supporting Information. Each cluster was annotated on the basis of canonical marker gene expression and subsequently mapped to one of the ten major cell types. This mapping enabled us to systematically deconstruct the composition of each cell type into its constituent subclusters and examine their dynamic shifts between the nmEV‐treated and control groups. The relative proportion and absolute number of nuclei in each cluster are shown in Figure 4B,C, respectively.

Through this refined resolution, we identified five clusters, Clusters 0, 6, 7, 10, and 20, which exhibited statistically significant alterations in abundance (P < 0.05), representing differentially regulated subpopulations responsive to nmEVs. These clusters were distributed among excitatory neurons (Cluster 0), inhibitory neurons (Clusters 6, 10, and 20), and astrocytes (Cluster 7). Interestingly, the three inhibitory neuronal clusters displayed divergent trends, with Clusters 6 and 10 significantly reduced and Cluster 20 markedly increased. In contrast, although the overall proportion of astrocytes decreased, Cluster 7 increased, suggesting a potentially reactive or functionally distinct astrocytic subset (Figure 4D).

These findings highlight the capacity of nmEVs to selectively remodel hippocampal cell‐type substructures, prompting us to further explore the functional significance of these subclusters in the context of aging and cognitive decline.

### 2.6 Selective Remodeling of Inhibitory Neuron Subclusters by nmEV Treatment

To investigate the potential biological functions of the differentially regulated inhibitory neuron subclusters, we performed Gene Ontology (GO) and Kyoto Encyclopedia of Genes and Genomes (KEGG) enrichment analyses of Clusters 6, 10, and 20. Clusters 6 and 10, both of which were significantly reduced following nmEV treatment, exhibited highly overlapping enrichment profiles. The GO terms in these clusters were predominantly associated with the regulation of neurotransmitter transport, synaptic signaling, and positive regulation of cell communication (Figure S2A, Supporting Information). KEGG analysis further revealed enrichment of genes related to lysosomal activity, endocytosis, Fc gamma R‐mediated phagocytosis, and immune‐related signaling pathways, such as osteoclast differentiation and platelet activation (Figure S2B, Supporting Information). These results suggest that Clusters 6 and 10 may represent metabolically active or inflammation‐prone inhibitory neuron subsets whose suppression may contribute to the anti‐inflammatory and homeostatic rebalancing effects of nmEVs in the aging hippocampus.

In contrast, Cluster 20, which was markedly increased upon nmEV administration, demonstrated a distinct enrichment profile, indicating enhanced neuronal functionality. GO terms related to the regulation of membrane potential, synaptic transmission, and neurotransmitter release were enriched (Figure S3A, Supporting Information). KEGG analysis revealed several neural communication‐ and rhythm‐related pathways, including glutamatergic and dopaminergic synapses, circadian entrainment, and oxytocin signaling (Figure S3B, Supporting Information). These findings suggest that Cluster 20 may represent a functionally active inhibitory neuronal subpopulation that is preferentially preserved or expanded by nmEVs, potentially contributing to improved synaptic integrity and circadian homeostasis during aging.

### 2.7 Functional Enrichment Analysis in Cluster 0 Reveals Multifaceted Neuroprotective Programs

Among all the clusters, Cluster 0 demonstrated the strongest transcriptomic response to nmEVs and was therefore selected as the primary target for in‐depth functional annotation. To elucidate the functional implications of the expanded excitatory neuron subpopulation (Cluster 0), we performed GO and KEGG enrichment analyses on its cluster‐specific marker genes. GO biological process enrichment revealed prominent activation of pathways involved in synaptic plasticity, neurotransmitter transport, neuron projection development, and oxidative stress response, all of which are highly relevant to the restoration of excitatory neuronal function (Figure 4E). Complementary KEGG enrichment revealed significant upregulation of pathways such as oxidative phosphorylation, carbon metabolism, and neurodegeneration‐associated signaling, indicating a simultaneous increase in energy metabolism, synaptic function, and cellular resilience (Figure 4F).

Notably, Cluster 0 was enriched in pathways related to antiapoptotic regulation (“positive regulation of cell survival,” “regulation of neuron death”) and cellular repair mechanisms, including “response to DNA damage stimulus” and “cellular response to oxidative stress.” These findings suggest that nmEVs not only increase excitatory neurotransmission but also promote a neuroprotective transcriptional state that may support long‐term neural circuit stability. Taken together, these results suggest that Cluster 0 represents a functionally enhanced subset of excitatory neurons that may contribute to the pro‐cognitive and neuroprotective effects of nmEVs in the aging hippocampus.

Furthermore, we performed cell–cell communication analysis to explore how representative clusters (Cluster 0 for excitatory neurons, Cluster 20 for inhibitory neurons, and Cluster 6 for astrocytes) interact with surrounding cell types (Figure S4A–C, Supporting Information). The data revealed increased outgoing and incoming signaling activities, including synaptic regulation, neurotrophin signaling, and inflammatory suppression. This integrative analysis supports a model in which nmEVs not only modulate internal gene expression programs but also reshape the intercellular signaling network to support neuronal survival and functional restoration.

### 2.8 Functional Profiling of the Expanded Glial Subcluster

Cluster 7, a glial subpopulation that was significantly expanded following nmEV treatment, was subjected to GO and KEGG enrichment analyses to elucidate its potential functional role. GO biological process terms were markedly enriched in pathways associated with intercellular signaling, including the positive regulation of cell communication, transsynaptic signaling, and neurotransmitter transport (Figure S5A, Supporting Information). These findings suggest that Cluster 7 may represent an astrocytic subpopulation actively involved in the modulation of synaptic transmission and neural circuit dynamics.

KEGG analysis revealed that genes in this cluster were significantly enriched in several neurotransmission‐related pathways, including glutamatergic, GABAergic, and dopaminergic synapses, as well as the calcium signaling pathway and retrograde endocannabinoid signaling (Figure S5B, Supporting Information). Notably, the circadian entrainment pathway was also enriched, which is consistent with a role for astrocytes in regulating brain rhythmicity. The expansion of this glial subcluster upon nmEV administration may indicate a neuroprotective remodeling process in which astrocytes contribute to restoring synaptic function and circadian balance in the aging hippocampus.

### 2.9 Molecular and Functional Validation of Single‐Cell Transcriptomic Findings

Cognitive decline is among the most prominent and debilitating manifestations of aging and is often linked to hippocampal dysfunction and disrupted neuronal connectivity.^[^ ^15^, ^16^ ^]^ To assess whether nmEVs could ameliorate age‐associated cognitive deficits, we used the Morris water maze (MWM), a well‐established behavioral paradigm used to evaluate spatial learning and memory in rodent models. This test is particularly sensitive to hippocampus‐dependent navigation and has been widely used to assess cognitive performance in aging and neurodegenerative disease models.

**Figure 5:**
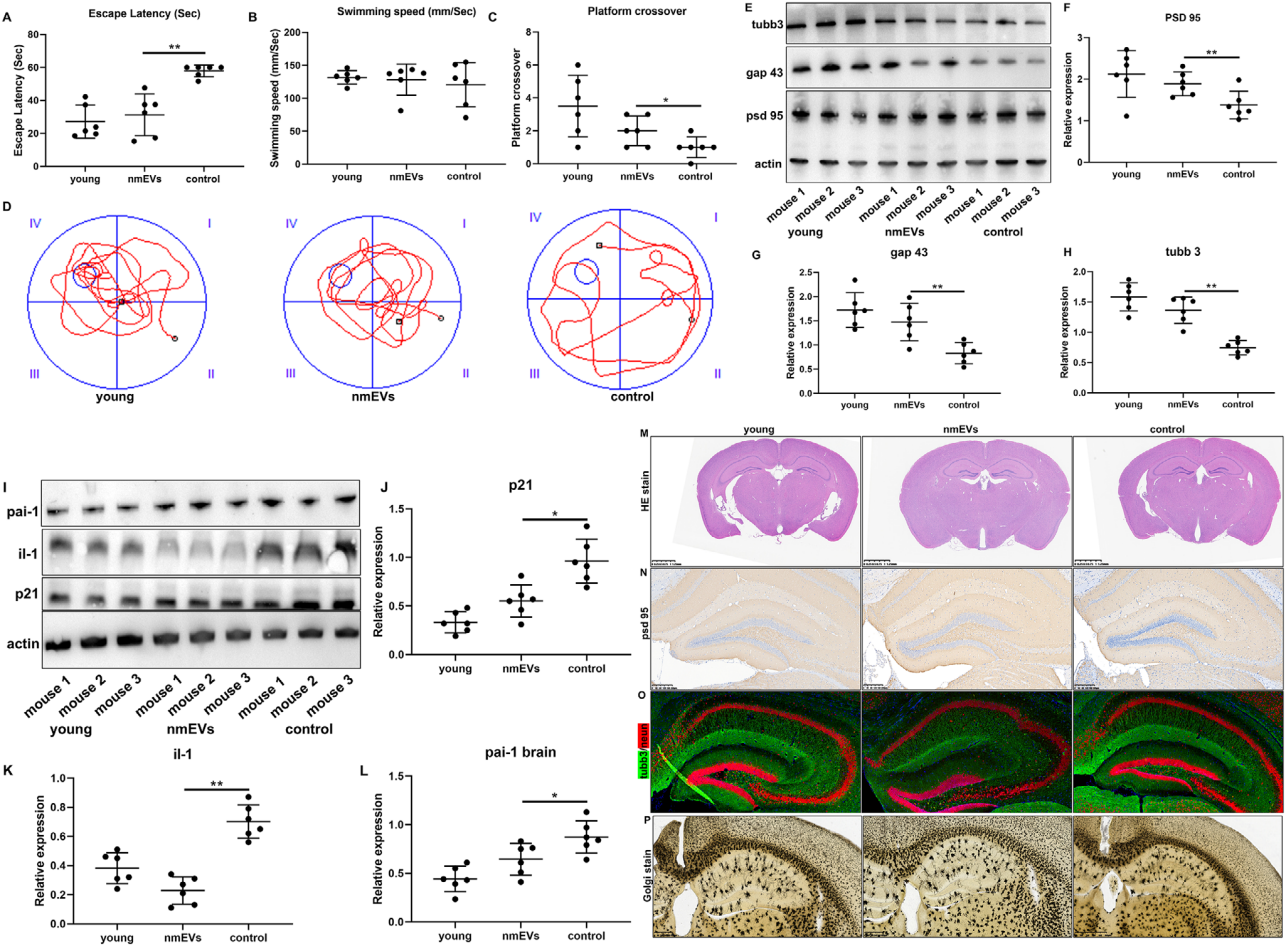
nmEVs improve cognition and suppress hippocampal senescence. A,B) Morris water maze (MWM) learning curves showing reduced escape latency in the nmEV group. C,D) Probe trial results indicating increased time spent in the target quadrant and platform crossings by nmEV‐treated mice. E–H) western blot analysis indicating increased expression of PSD95, GAP43, and TUBB3 after nmEV treatment. I–L) Western blot analysis indicating reduced IL‐1, PAI‐1, and p21 levels in the hippocampus following treatment. M–P) Histological and immunohistochemical staining confirming the improved neuronal architecture, dendritic complexity, and synaptic integrity in nmEV‐treated mice. Data are presented as the mean ± SD; n = 6 biological replicates per group. Data are presented as mean ± SD (n = 6 biological replicates per group). Statistical significance was determined using one‐way ANOVA followed by Tukey's post‐hoc test for multiple group comparisons, or unpaired two‐tailed *t*‐test for pairwise comparisons, as appropriate. *p < 0.05, **p < 0.01.

Compared with PBS‐treated control mice, nmEV‐treated aged mice exhibited significantly improved spatial learning and memory performance. During the acquisition phase, the escape latency of nmEV‐treated mice markedly decreased across training days, indicating enhanced learning ability (**Figure** **5**A,B). In the probe trial, these mice spent significantly more time in the target quadrant and crossed the former platform location more frequently, suggesting improved memory retention (Figure 5C). Notably, the performance of nmEV‐treated aged mice approached that of young adult controls, highlighting the potential of nmEVs to reverse age‐related cognitive impairment (Figure 5D). These behavioral improvements corroborated the molecular insights obtained from single‐nucleus RNA sequencing, collectively confirming that nmEVs effectively alleviate aging‐related cognitive decline.

To validate the transcriptional predictions, we conducted a series of protein‐level and morphological experiments. Western blot analysis confirmed that the expression of psd 95, gap 43, and tubb3, which were all involved in the top enriched GO/KEGG pathways (synaptic plasticity, axonogenesis, and neurostructural remodeling) (Figure 5E), was significantly upregulated in the hippocampal tissue of nmEV‐treated mice compared with that in the PBS‐treated aged mice (Figure 5F–H). These findings directly support the transcriptomic indications of increased synaptic activity and neuronal plasticity in excitatory neurons. In parallel, we examined the expression of canonical senescence‐associated markers in the hippocampus. Western blot analysis revealed that nmEV treatment markedly suppressed the protein levels of il‐1, pai‐1, and p21 (Figure 5I), which are hallmark components of the senescence‐associated secretory phenotype (SASP), compared to those in aged controls (Figure 5J–L). These results further demonstrate that nmEVs not only promote neuronal remodeling but also alleviate local cellular senescence in the aging brain.

Further histological evaluation revealed consistent results. Hematoxylin‒eosin (HE) staining revealed improved cytoarchitectural integrity of the hippocampus in the nmEV group (Figure 5M). Immunohistochemical staining for PSD95 (Figure 5N) and dual immunofluorescence staining for tubb 3 and NeuN further confirmed the increased presence of structurally and functionally mature neurons following nmEV treatment (Figure 5O). Importantly, Golgi staining revealed increased dendritic arborization and spine‐like projections, which were consistent with the restoration of neuronal connectivity (Figure 5P).

Importantly, the molecular and cellular findings obtained through single‐nucleus RNA sequencing and subsequent immunohistochemical validation provide mechanistic insights into the behavioral improvements observed in aged mice following nmEV treatment. As shown by the results of the Morris water maze test, nmEV‐treated mice exhibited significantly enhanced spatial learning and memory capabilities, indicating the restoration of cognitive function. The observed improvements in excitatory synaptic gene expression, neuronal structural proteins (e.g., psd 95, gap 43, and tubb 3), and dendritic complexity (via Golgi staining), as well as reductions in glial reactivity and the restoration of cell cycle activity, suggest that nmEVs enhance hippocampal circuit plasticity and functional integrity. These molecular changes are likely to underlie the behavioral rescue observed in the water maze, highlighting a strong correlation among transcriptomic remodeling, neuroanatomical recovery, and cognitive performance.

Taken together, these results suggest that nmEVs represent a promising intervention capable of reversing age‐related cognitive decline through coordinated molecular, structural, and behavioral modulation.

### 2.10 Global Transcriptomic Insights into the Rejuvenating Potential of nmEVs

Aging is a systemic process that affects nearly all organs and tissues, leading to a progressive decline in physiological integrity and functional coordination across the body.^[^ ^17^, ^18^ ^]^ Accumulating evidence suggests that aging not only drives organ‐specific dysfunction but also disrupts interorgan communication, contributing to the emergence of complex, multimorbid phenotypes in elderly individuals.^[^ ^19^, ^20^ ^]^ To systematically evaluate the biological mechanisms underlying the antiaging effects of nasal nmEVs, we conducted transcriptomic profiling of five major aging‐sensitive organs, namely, the heart, liver, spleen, lung, and kidney. Given the systemic nature of aging, which involves both intrinsic cellular decline and disrupted interorgan signaling, this multiorgan approach enables a comprehensive understanding of nmEV‐mediated molecular remodeling.

To identify robust and reproducible molecular alterations, we applied a cross‐organ enrichment strategy. Only KEGG pathways or GO‐BP terms that were significantly enriched (adjusted p < 0.05) and appeared in three or more organs were considered biologically relevant. This filtering allowed us to capture system‐wide regulatory signatures associated with nmEV treatment.

**Figure 6:**
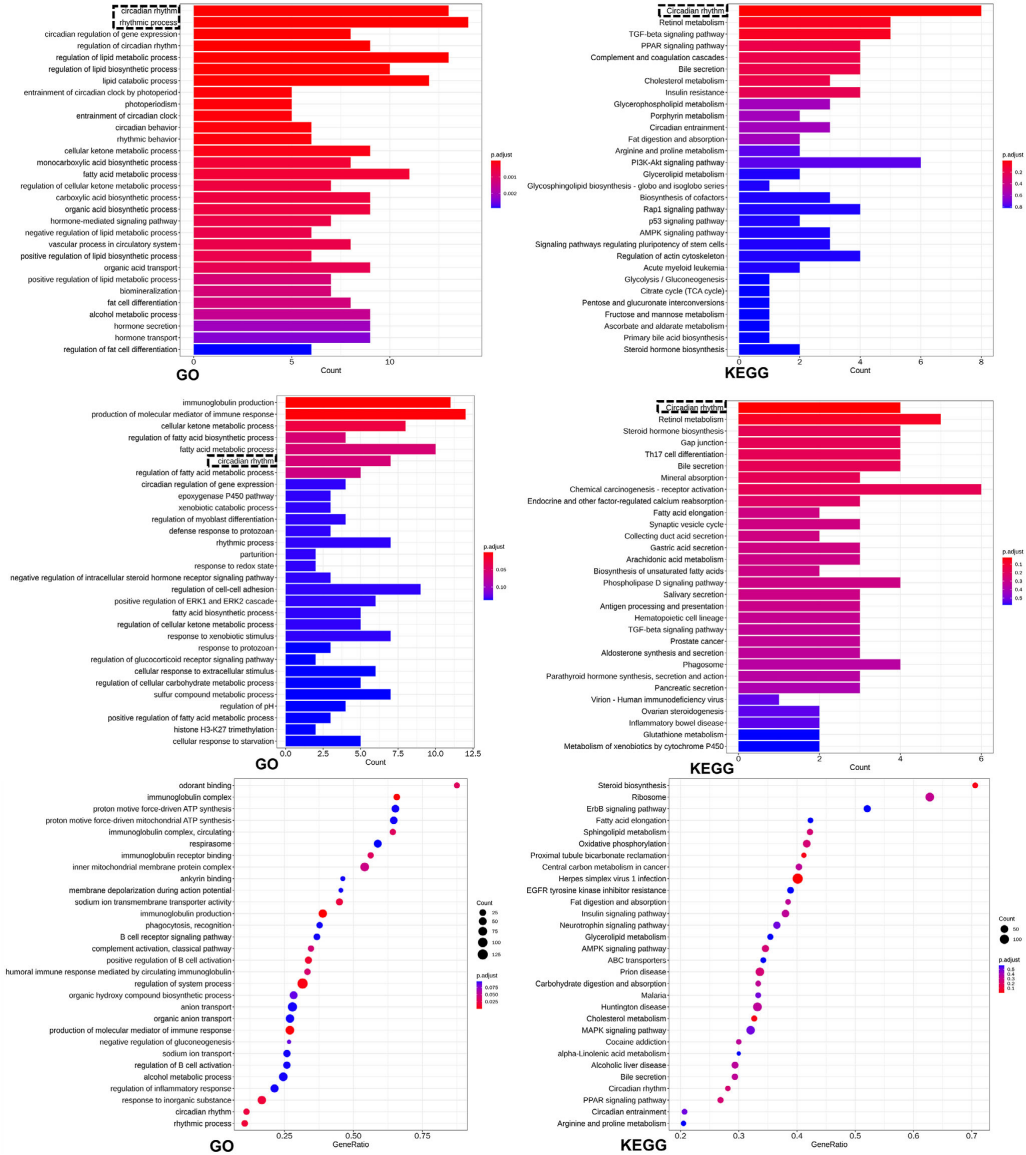
Cross‐organ transcriptomics reveal circadian and immune pathway reprogramming by nmEVs. A,B) GO and KEGG enrichment in the liver showing suppression of circadian rhythm pathways. C,D) Upregulated GO and KEGG terms indicating enhancement of immune surveillance and cytokine signaling. E,F) gene set enrichment analysis (GSEA) plots showing coordinated downregulation of circadian rhythm and p53 apoptotic signaling in liver tissue. Transcriptomic analysis was performed on RNA extracted from liver tissues of three biological replicates per group (n = 3). Enrichment statistics were calculated using adjusted p values (Benjamini–Hochberg correction); data are presented as normalized enrichment scores (NES) with false discovery rate thresholds.

The downregulated transcriptomic programs highlighted two dominant mechanistic axes. First, circadian rhythm‐related pathways, including both KEGG (circadian rhythm and circadian entrainment) and GO‐BP (circadian regulation of gene expression, entrainment of circadian clock, and rhythmic processes) terms, were consistently suppressed across the heart, liver, lung, and kidney. We present the GO (**Figure** **6**A) and KEGG (Figure 6B) enrichment results from the liver as a representative example. Enrichment results from the remaining organs are provided in Figures S6–S9, Supporting Information for reference. These findings indicate that nmEVs may reset age‐disrupted biological rhythms, restoring molecular synchrony across tissues. Second, cellular senescence, a hallmark of aging, was notably downregulated, especially in the heart and spleen. These findings suggest that nmEVs may mitigate DNA damage responses, reduce prosenescence signaling, and limit SASP activity, thereby contributing to tissue rejuvenation.

In contrast, the upregulated gene sets revealed a distinct but complementary trend. GO‐BP terms such as immunoglobulin production, humoral immune response, and complement activation were enriched in multiple organs, reflecting enhanced B‐cell function and innate immune readiness. In parallel, KEGG pathways, including cytokine–cytokine receptor interaction, NF‐κB signaling, and JAK‐STAT signaling, were also upregulated, indicating the activation of structured immune regulatory networks. Rather than representing chronic inflammation, these changes likely constitute an adaptive immune reconfiguration, characterized by improved immune surveillance, tissue repair, and the restoration of immune homeostasis, which are hallmarks of healthy aging. We present the GO (Figure 6C) and KEGG (Figure 6D) enrichment results from the liver as a representative example. Enrichment results from the remaining organs are provided in Figures S10–S13, Supporting Information for reference.

Together, these findings support a dual‐mechanism model through which nmEVs exert systemic antiaging effects by (1) reprogramming tissue‐specific circadian dysregulation and (2) remodeling immune function toward a regenerative phenotype. This integrated modulation of timekeeping and immunity underscores the potential of nmEVs as a multifaceted intervention for age‐associated physiological decline.

### 2.11 GSEA Reveals Coordinated Modulation of Aging Pathways and Systemic Suppression of p53‐Mediated Aging

To further validate and complement the pathway‐level findings derived from classical GO and KEGG enrichment analyses, we applied gene set enrichment analysis (GSEA) across five key organs, namely, the heart, liver, spleen, lung, and kidney. Unlike conventional methods that rely on predefined thresholds for differential gene expression, GSEA leverages ranked gene lists to capture subtle yet coordinated shifts in gene sets, making it particularly suitable for dissecting complex and distributed biological processes such as aging.^[^ ^21^ ^]^

Consistent with our earlier findings, GSEA revealed that circadian rhythm‐related pathways, including those related to the entrainment of the circadian clock, circadian regulation of gene expression, and regulation of rhythmic processes, were negatively enriched in multiple organs, especially the heart and kidney. This convergence across analytical methods reinforces the interpretation that nmEVs may restore temporal coherence and peripheral clock function in aged tissues. These results not only support the robustness of our GO and KEGG analyses but also highlight the ability of GSEA to detect systemic biological reprogramming beyond statistical cutoff values. The results of the GSEA are shown for the liver as a representative organ (Figure 6E,F). Full GSEA enrichment profiles for the heart, spleen, lung, and kidney are provided in Figures S14–S17, Supporting Information.

In addition, GSEA revealed a range of hallmark‐related biological processes commonly implicated in aging, including the downregulation of mitochondrial translation, protein homeostasis, and telomere‐associated pathways, as well as the upregulation of immune activation, ECM organization, and nutrient‐sensing networks. Among these pathways, p53 signaling emerged as one of the most consistently and broadly altered aging‐related pathways across all five organs. The GO term regulation of intrinsic apoptotic signaling pathway by p53 class mediator was negatively enriched in every tissue examined, suggesting systemic suppression of p53‐mediated apoptosis following nmEV treatment.

This consistent reduction in p53 apoptotic signaling likely reflects a biological rebalancing away from the chronic p53 overactivation observed in aged tissues, which is often associated with excessive cellular senescence, inflammation, and functional decline. The dampening of p53 activity may therefore represent a key molecular axis through which nmEVs exert their tissue‐protective and antiaging effects.

### 2.12 Experimental Validation of Antiaging Effects in the Liver, Lung, Spleen, and Kidney

Aging induces a spectrum of pathological alterations across multiple organs, including the kidney, liver, spleen, and lung, which are particularly susceptible to chronic inflammation, fibrosis, and functional decline.^[^ ^22^ ^]^ These tissues collectively represent key nodes of systemic homeostasis, and their parallel deterioration reflects the widespread impact of organismal aging. Their selection in this study not only captures diverse aging phenotypes but also allows for rigorous evaluation of therapeutic efficacy across structurally and functionally distinct systems. Furthermore, this multiorgan focus builds upon our prior transcriptomic profiling and enables validation of key molecular signatures at the tissue level, reinforcing their translational relevance. These organs were thus selected for subsequent histological and molecular analysis to assess the rejuvenating potential of nmEVs to reverse age‐associated degeneration.

**Figure 7:**
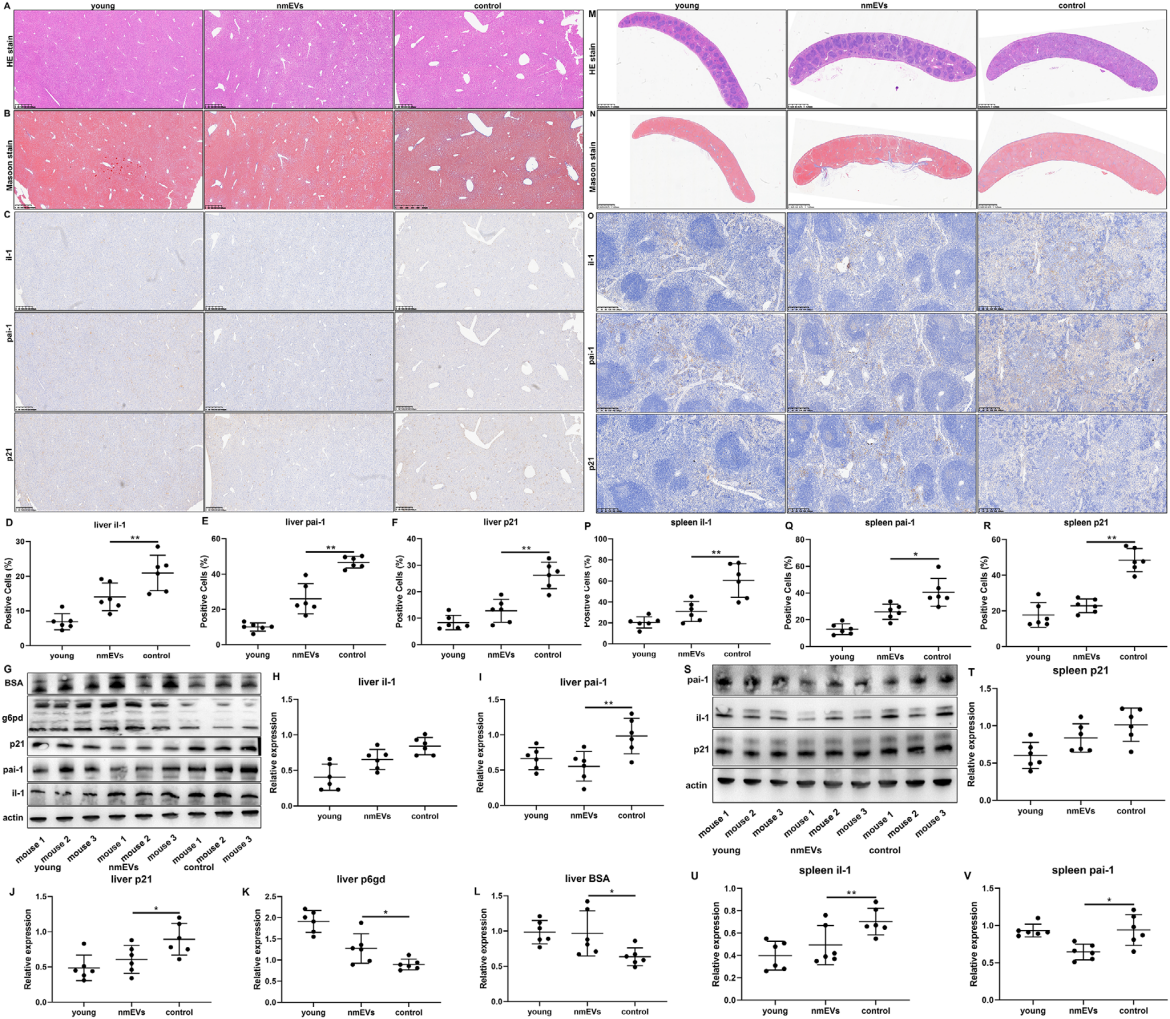
Histological and molecular validation of the antiaging effects in the liver and spleen. A,B) H&E and Masson's staining showing reduced inflammation and fibrosis in the liver after treatment. C–F) IHC for IL‐1, PAI‐1, and p21 confirming reduced SASP marker expression. G–L) Western blotting was used to quantify SASP suppression and functional marker upregulation (BSA and G6PD) in the liver. M–R) Histological and IHC analyses of the spleen demonstrating decreased fibrosis, hemosiderin, and senescence marker expression in nmEV‐treated mice. S–V) Western blot analysis corroborating reduced IL‐1β, PAI‐1, and p21 levels in the spleen. Data are presented as mean ± SD (n = 6 biological replicates per group). Statistical significance was determined using one‐way ANOVA followed by Tukey's post‐hoc test for multiple group comparisons, or unpaired two‐tailed *t*‐test for pairwise comparisons, as appropriate. *p < 0.05, **p < 0.01.

In the liver, histological evaluation across the three experimental groups revealed notable age‐related structural changes and improvements following nmEV intervention. While the general lobular architecture remained intact in all groups according to H&E staining, aged livers showed disorganized hepatic cords, hepatocyte shrinkage, nuclear pyknosis, and prominent lipofuscin accumulation with inflammatory infiltration (**Figure** **7**A). In contrast, nmEV‐treated livers exhibited more orderly hepatocyte arrangement, reduced nuclear condensation, and attenuated lipofuscin and inflammatory signals. Masson's trichrome staining confirmed marked collagen accumulation in the aged group, particularly around central veins and portal triads, which formed fibrous septa and bridging fibrosis (Figure 7B). This was markedly reduced in the nmEV group, suggesting attenuation of hepatic fibrosis. Immunohistochemical analysis of liver tissues revealed that the expression of il‐1, pai‐1, and p21 was markedly lower in nmEV‐treated mice than in aged control mice (Figure 7C). Quantitative assessment revealed a significant decrease in the il‐1‐, pai‐1‐, and p21‐positive cell populations (Figure 7D–F).

To assess whether nmEVs attenuate this senescent‐inflammatory axis, we next examined the expression of these SASP markers in liver tissue by western blotting. To further validate these observations, western blot analysis was performed (Figure 7G). Key SASP‐related proteins, including il‐1, pai‐1, and p21, were notably decreased in the nmEV group, reinforcing the anti‐senescence effect (Figure 7H–J). Interestingly, we also observed increased expression of BSA and g6pd (Figure 7K,L), two functional markers of hepatocellular biosynthetic activity. These findings suggest not only a reduction in aging burden but also a potential restoration of hepatic functional capacity. This molecular improvement was consistent with decreases in plasma AST and ALT levels, further supporting the conclusion that nmEVs improve both the structure and function of aging liver tissue.

In the spleen, age‐associated morphological and molecular alterations were similarly observed. H&E staining revealed structural disorganization in aged mice, including blurred boundaries between red and white pulp, increased hemosiderin deposition, and mild lymphocyte depletion, which are hallmarks of splenic aging (Figure 7M). In contrast, the histoarchitecture of the spleens from nmEV‐treated mice was more preserved, with clearer red–white pulp demarcation, reduced hemosiderin accumulation, and improved lymphoid cellularity, indicating attenuated age‐related degeneration. Masson's trichrome staining revealed excessive collagen deposition in the splenic capsule and perivascular areas of aged controls, reflecting fibrotic remodeling associated with aging. This accumulation was significantly reduced in the nmEV group, as evidenced by a decrease in red‐stained regions, suggesting that the extracellular matrix balance and tissue integrity were restored (Figure 7L).

Immunohistochemically, the expression of the senescence‐ and SASP‐associated proteins il‐1β, pai‐1, and p21 was markedly elevated in the aged control group (Figure 7O). Following nmEV treatment, these markers were substantially downregulated in terms of both spatial distribution and intensity (Figure 7P–R). Western blot analysis further validated this trend (Figure 7S), revealing significant reductions in the protein levels of il‐1β, pai‐1, and p21 in nmEV‐treated spleens compared with those in aged control spleens (Figure 7T–V). Collectively, these findings indicate that nmEVs alleviate splenic inflammation and cellular senescence, contributing to the preservation of splenic structure and immunological function during aging.

In the lung, age‐related structural and molecular alterations were also evident. H&E staining of lung tissue from aged mice revealed thickened alveolar walls, narrowed airspaces, and mild interstitial inflammatory infiltration, which is consistent with typical aging pathology. In contrast, nmEV‐treated lungs exhibited a more preserved alveolar architecture, reduced interstitial thickening, and attenuated inflammatory cell presence (Figure S18A, Supporting Information). Masson's trichrome staining demonstrated increased collagen deposition in aged lung tissues, particularly in the peribronchial and interalveolar regions. This fibrotic remodeling was markedly reduced in the nmEV‐treated group, with smaller red‐stained areas, indicating the reversal of age‐associated ECM accumulation (Figure S18B, Supporting Information). Immunohistochemically, il‐1β, pai‐1, and p21 expression was increased in the aged control group but significantly decreased following nmEV intervention (Figure S18C, Supporting Information). Western blot analysis confirmed that expression of the SASP‐related proteins il‐1β, pai‐1, and p21 was downregulated in the nmEV group (Figure S18D–G, Supporting Information). These molecular changes support the histological evidence of reduced cellular senescence and inflammatory burden, further suggesting that nmEVs can ameliorate structural aging and promote tissue homeostasis in the lung.

In the kidney, aging was associated with tubular epithelial atrophy, glomerulosclerosis, and increased interstitial fibrosis, as shown by H&E staining. The kidneys of aged control mice exhibited a disorganized renal architecture, reduced tubular volume, and inflammatory infiltrates, whereas those of the nmEV‐treated group exhibited improved tubular structure, decreased interstitial space, and reduced inflammatory features (Figure S18H, Supporting Information). Masson staining revealed extensive peritubular and periglomerular collagen accumulation in aged kidneys, resulting in the formation of fibrotic bands that were markedly reduced in the nmEV group (Figure S18I, Supporting Information). Immunohistochemical analysis revealed reduced il‐1β, pai‐1, and p21 expression, particularly around glomeruli and renal vasculature, indicating both antifibrotic and anti‐senescence effects of nmEVs (Figure S18J, Supporting Information). Western blotting further confirmed decreases in the expression of il‐1β, pai‐1, and p21, along with the preserved expression of renal functional proteins, suggesting that nmEVs not only reduce the senescence burden but also help restore kidney tissue integrity and function (Figure S18K–M, Supporting Information).

Collectively, the results of the histological, immunohistochemical, and molecular analyses across the liver, lung, and kidney revealed consistent trends: nmEV treatment attenuated tissue fibrosis, reduced cellular senescence, and promoted an anti‐inflammatory tissue environment. These findings provide strong experimental support for the transcriptomic predictions, highlighting nmEVs as a multiorgan rejuvenation strategy targeting key aging mechanisms.

### 2.13 nmEVs Induce Circadian Resynchronization in Aged Organs

The circadian rhythm is a fundamental regulatory mechanism that governs tissue homeostasis, metabolism, and immune responses in a time‐of‐day‐dependent manner.^[^ ^23^, ^24^ ^]^ Disruption of the circadian rhythm has been increasingly recognized as a contributor to aging and age‐related diseases, particularly in organs such as the liver, kidney, and lung, where temporal regulation of function is critical for maintaining physiological balance.^[^ ^25^, ^26^ ^]^ Aging has been shown to impair the expression and coordination of core clock components, leading to metabolic inflexibility, chronic inflammation, and tissue dysfunction.^[^ ^27^, ^28^ ^]^ In this study, transcriptomic profiling of these organs revealed a consistent downregulation of circadian rhythm‐associated pathways, including circadian regulation of gene expression and entrainment of the circadian clock, suggesting that aging in these tissues may be accompanied by molecular desynchronization of peripheral clocks.

**Figure 8:**
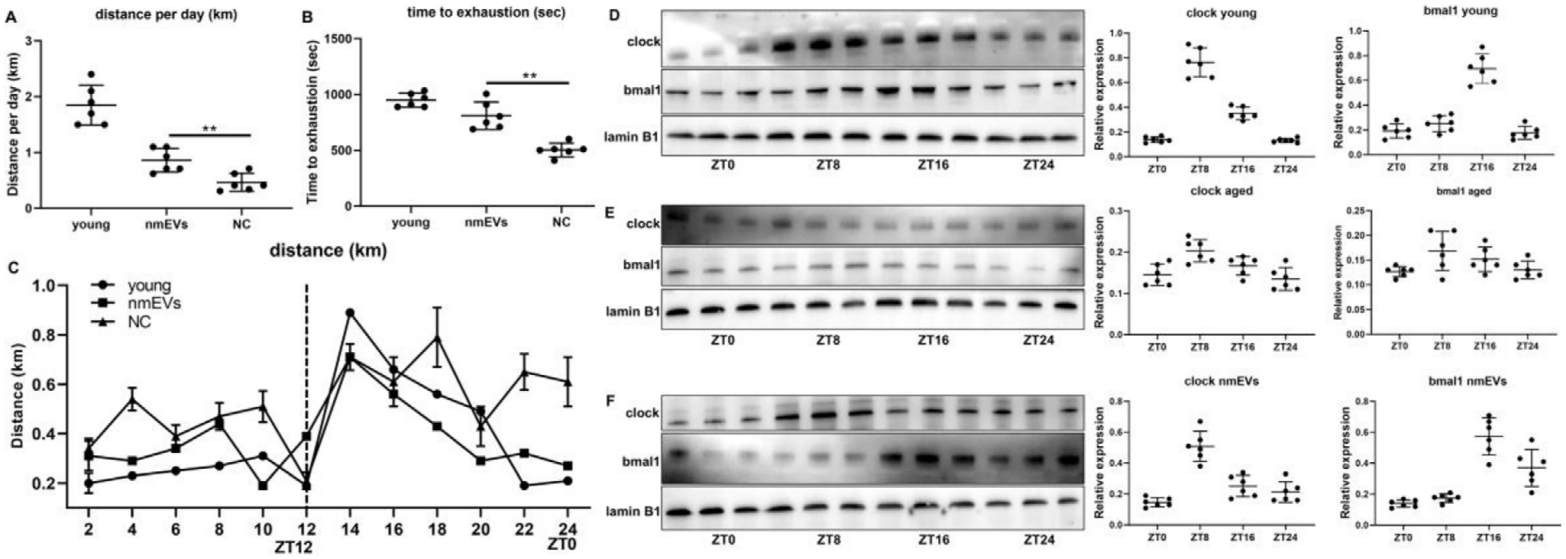
nmEVs restore circadian rhythm in aged mice at the behavioral and molecular levels. A,B) Voluntary wheel running and treadmill exhaustion tests demonstrating improved physical activity. C) Hourly activity patterns showing restored light‐phase activity preference in the nmEV group. D–F) Western blot analysis of liver nuclear extracts at ZT0/8/16/24 showing rescued Clock and Bmal1 expression rhythms in treated mice. Data are presented as mean ± SD (n = 6 biological replicates per group). Statistical significance was determined using one‐way ANOVA followed by Tukey's post‐hoc test for multiple group comparisons, or unpaired two‐tailed *t*‐test for pairwise comparisons, as appropriate. *p < 0.05, **p < 0.01.

To functionally assess whether nmEVs influence behavioral circadian rhythmicity and physical performance in aged mice, we conducted a series of voluntary wheel running and treadmill exhaustion tests coupled with light/dark cycle activity monitoring. Compared with aged controls, nmEV‐treated mice exhibited a significant increase in daily voluntary running distance and increased treadmill time to exhaustion, indicating improved endurance capacity and overall physical vitality (**Figure** **8**A,B). Hourly analysis of voluntary wheel running activity revealed distinct differences in circadian behavior patterns between groups. In aged control mice, wheel running was distributed irregularly across the 24‐h cycle, with no clear preference for either the light (ZT0–ZT12) or dark (ZT12–ZT24) phase, indicating a disrupted or desynchronized circadian rhythm (Figure 8C). This pattern reflects behavioral manifestations of age‐related circadian dysfunction in which animals may exhibit fragmented activity and impaired sleep‒wake coordination.

In contrast, nmEV‐treated aged mice exhibited a reorganized running pattern resembling that of young mice. Specifically, treated animals showed increased voluntary activity during the light period (ZT0–ZT12) and reduced activity during the dark phase, suggesting a partial realignment of rest–activity cycles toward a more youthful and synchronized profile. This normalization of behavior supports the notion that nmEVs contribute to circadian rhythm restoration at both the molecular and functional levels. These findings indicate that nasal mucosa‐derived EVs can improve physical function and biochemical homeostasis in aged mice, supporting their potential as a systemic antiaging intervention.

To further investigate whether nmEVs restore circadian rhythmicity at the molecular level, we performed temporal profiling of core clock proteins in the liver across a 24‐h cycle. Mice were sacrificed at ZT0, ZT8, ZT16, and ZT24, and nuclear extracts from liver tissues were analyzed via western blotting to quantify Clock and Bmal1 expression dynamics.

In young control mice, Clock displayed a rhythmic nuclear expression pattern characterized by a pronounced peak at ZT8 followed by a gradual decline. Bmal1 nuclear levels subsequently peaked at ZT16, reflecting the expected phase‐shifted oscillatory relationship between these two core circadian regulators (Figure 8D). This sequential expression confirms intact circadian rhythmicity at the molecular level. In contrast, aged mice exhibited a blunted expression profile, with both Clock and Bmal1 remaining at relatively constant low nuclear levels across all time points, suggesting a loss of temporal regulation and circadian collapse in the aged liver (Figure 8E). Strikingly, aged mice treated with nmEVs showed a reemergence of temporal dynamics: Clock nuclear expression peaked at ZT8, and Bmal1 expression peaked at ZT16, partially recapitulating the rhythmic pattern observed in young controls (Figure 8F).

These molecular findings were consistent with the behavioral circadian restoration observed in the voluntary wheel‐running assay, supporting the notion that nmEVs can reprogram circadian oscillations at both the behavioral and molecular levels in aged organisms.

### 2.14 nmEVs Ameliorate Senescence In Vitro

Stem cell exhaustion is a recognized hallmark of aging and is characterized by diminished self‐renewal, impaired differentiation capacity, and increased senescence of adult stem cell populations.^[^ ^29^, ^30^ ^]^ In the context of skeletal aging, BMSCs play a critical role in maintaining bone homeostasis through osteogenic support, immunomodulation, and repair functions. However, BMSCs from aged individuals exhibit functional decline, contributing to impaired bone regeneration and systemic frailty.

To assess the anti‐senescence effects of nmEVs in a clinically relevant model, we initially considered the use of BMSCs derived from aged mice. However, given that our therapeutic nmEVs were isolated from human nasal mucosa, we sought to avoid potential interspecies discrepancies and increase their translational value. Thus, we isolated human BMSCs from femoral heads obtained during hip arthroplasty in elderly patients with femoral neck fractures, providing a physiologically and clinically relevant model to test the effects of nmEVs on human stem cell aging in vitro. This strategy allowed us to directly evaluate the therapeutic potential of nmEVs in rejuvenating senescent hBMSCs and restoring their regenerative phenotype, offering insights into future clinical applications in aging‐related disorders.

To investigate the anti‐senescence effects of nmEVs at the cellular level, we established an in vitro model with hBMSCs isolated from elderly donors undergoing hip arthroplasty. Initial characterization confirmed the identity and multipotency of the hBMSCs, as shown by high expression of canonical mesenchymal surface markers, including CD44 and CD105, but not CD14 (Figure S19A, Supporting Information). We observed that these cells rapidly entered a senescent state by passage 5, as evidenced by decreased proliferative capacity (Figure S19B, Supporting Information) and a significant increase in the number of SA‐β‐gal‐positive cells (Figure S19C, Supporting Information).

To test the functional effects of nmEVs, we labeled and administered nmEVs to senescent hBMSCs. Confocal imaging confirmed efficient internalization of nmEVs by hBMSCs (Figure S20, Supporting Information). Following treatment with nmEVs for 48 h, western blot analysis revealed that the expression levels of key senescence‐associated proteins, including p21, il‐1, p53 and p16 (Figure S21A, Supporting Information), were significantly reduced compared with those in the untreated controls (Figure S21B–E, Supporting Information). No further enhancement was observed between the 48‐h treatments. In parallel, β‐gal staining revealed a marked reduction in the proportion of β‐gal‐positive cells upon nmEV treatment, indicating a reversal of the senescent phenotype at the cellular level (Figure S21F, Supporting Information).

In addition to reversing classical markers of cellular senescence, we further evaluated whether nmEVs could restore the osteogenic differentiation potential of senescent hBMSCs, a key functional deficit associated with aging. hBMSCs were pretreated with nmEVs, followed by osteogenic induction. ALP staining, performed after 7 days, revealed a significant increase in the ALP‐positive area in the nmEV‐treated group, indicating increased early osteogenic commitment (Figure S21G, Supporting Information). Moreover, ARS staining, which was conducted on Day 21, revealed more extensive mineralized nodule formation in the nmEV group, suggesting improved late‐stage osteogenic maturation (Figure S21H, Supporting Information). At the molecular level, western blot analysis confirmed the upregulation of the expression of two key osteogenic markers, OPN and Osx, in response to nmEV treatment (Figure S21I–K, Supporting Information). These results collectively demonstrate that nmEVs rejuvenate the osteogenic capacity of aged hBMSCs, supporting their potential use in bone regeneration therapies for aging populations.

Taken together, these findings indicate that nmEVs effectively ameliorate multiple hallmarks of cellular senescence in hBMSCs, including SASP suppression, proliferation recovery, and oxidative stress reduction, supporting their therapeutic potential in human stem cell aging.

### 2.15 nmEVs Restore Circadian Rhythmicity in Aging Cells

To determine whether nmEVs can restore intrinsic circadian rhythmicity in senescent hBMSCs, we first synchronized the cellular clocks in passage 5 cells with a serum shock protocol (50% FBS for 2 h), followed by treatment with nmEVs. Cells were subsequently fixed at four ZT points, ZT0, ZT8, ZT16, and ZT24, for immunofluorescence staining of Bmal1 and Clock.

In young hBMSCs (passage 2), robust circadian oscillation was observed. Notably, Clock showed distinct nuclear translocation at ZT8, followed by peak nuclear accumulation of Bmal1 at ZT16, which is consistent with their expected phase relationship in functional circadian feedback loops. In nmEV‐treated senescent hBMSCs, a similar oscillatory pattern was restored. Clock localized predominantly to the nucleus at ZT8 (Figure S21L, Supporting Information), and Bmal1 translocated into the nucleus at ZT16 (Figure S21M, Supporting Information), albeit with slightly reduced fluorescence intensity compared with that in young cells, indicating partial recovery of rhythmic dynamics. In contrast, control senescent hBMSCs (treated with PBS) displayed no evident nuclear‒cytoplasmic translocation of either Clock or Bmal1 at any time point. Both proteins remained diffusely distributed and lacked temporal fluctuation, reflecting a loss of circadian coordination.

These results suggest that nmEVs can effectively reinstate core molecular oscillations in aged hBMSCs, restoring the temporal compartmentalization of circadian regulators that is disrupted during cellular aging.

### 2.16 p53 Signaling Mediates the Restoration of Circadian Rhythm by nmEVs in hBMSCs

Building on our transcriptomic findings that nmEV treatment downregulated p53 signaling across multiple aging organs in vivo, we next investigated whether p53 modulation is mechanistically involved in the circadian rhythm restoration observed in senescent hBMSCs.

To assess this, we first synchronized passage 5 hBMSCs by serum shock and treated them with nmEVs in the presence or absence of a p53 stabilizer. Cells were fixed at key circadian time points and subjected to immunofluorescence staining for Clock and Bmal1. In the nmEV‐only group, we observed a characteristic oscillatory nuclear translocation pattern: Clock exhibited prominent nuclear localization at ZT8 (Figure S21N, Supporting Information), followed by Bmal1 nuclear enrichment at ZT16 (Figure S21O, Supporting Information). However, in the presence of the p53 stabilizer, this rhythmic nuclear redistribution was markedly inhibited. Both Clock and Bmal1 failed to show clear temporal nuclear accumulation, suggesting that persistent p53 activation suppresses circadian dynamics at the protein localization level.

These findings, combined with our observations that p53 stabilization disrupts rhythmic protein oscillation in otherwise young rhythmic hBMSCs, strongly indicate that p53 functions as a key upstream suppressor of cellular circadian rhythm. This was further evidenced by immunofluorescence analysis, which revealed that Bmal1 (Figure S22, Supporting Information) and Clock (Figure S23, Supporting Information) failed to exhibit nuclear translocation or rhythmic fluctuation across time points in the presence of p53 stabilization. Taken together, these findings suggest that the beneficial effects of nmEVs on circadian restoration depend, at least in part, on the downregulation or attenuation of p53 signaling.

## 3 Discussion

Aging is the single greatest risk factor for most chronic diseases that negatively affect health span and quality of life among older adults.^[^ ^31^ ^]^ In recent decades, substantial progress has been made in elucidating the biological underpinnings of aging, leading to the identification of several hallmarks and molecular drivers. Processes such as stem cell exhaustion, chronic inflammation, oxidative stress, mitochondrial dysfunction, and telomere attrition have been implicated in both systemic aging and the pathogenesis of age‐related diseases.

As aging progresses, these alterations manifest differently across tissues and organ systems, leading to distinct clinical phenotypes^[^ ^32^ ^]^ such as cognitive decline associated with neuronal aging or bone loss due to impaired osteogenic renewal. These diverse manifestations underscore the organ‐specific yet systemic nature of aging, posing a significant challenge to single‐target interventions. Consequently, regenerative strategies capable of orchestrating multidimensional repair and functional rebalancing across multiple biological systems are needed.

In most current studies, EVs are derived from a single, well‐defined cell type, such as BMSCs, adipose‐derived stem cells, or neural stem cells (NSCs).^[^ ^33^ ^]^ This reductionist approach offers clear advantages: it allows for the precise delineation of EV cargo, reduces biological variability, and facilitates mechanistic dissection without confounding effects. Indeed, single‐source EVs may show pronounced efficacy in organ‐specific aging conditions, such as BMSC‐derived EVs for osteoporosis or NSC‐derived EVs for Alzheimer's disease.^[^ ^34^ ^]^ These approaches have demonstrated considerable therapeutic value, particularly when aging‐associated dysfunction is localized, offering a highly targeted and disease‐specific intervention strategy that resembles organ‐level precision therapy. However, aging is a uniquely complex physiological‒pathological process that involves multiple organs, diverse cell populations, and systemic molecular deregulation. As such, it is questionable whether EVs from a single cell source can provide sufficient breadth of action to meaningfully counteract aging at the organismal level.

In this context, our study proposed a novel paradigm: leveraging EVs derived from a tissue characterized by rapid regeneration, minimal senescence burden, and an origin from multiple developmental lineages—the human nasal mucosa. We hypothesized that such EVs, which originate from a physiologically resilient and heterogeneously regenerative environment, may exert multifaceted antiaging effects, offering systemic benefits beyond localized tissue repair. We systematically demonstrated that nmEVs possess remarkable antiaging potential and are capable of improving multiple physiological functions in aged mice. Following repeated intravenous administration, treated animals exhibited enhanced physical condition, improved fur regeneration, and overall signs of rejuvenation compared to those of aged control animals. Among various tissues, the brain received particular attention, as nmEVs significantly improved cognitive performance, suggesting their benefits to the central nervous system. Beyond neurocognitive outcomes, we extended our investigation to five major organs, namely, the heart, liver, spleen, lung, and kidney, representing a cross‐section of systemic aging. Through transcriptomic profiling, histological assessment, and protein‐level validation, we revealed that nmEVs exert organ‐wide protective effects primarily by modulating the circadian rhythm and related pathways, a regulatory axis that has emerged as a key node in aging biology. Moreover, compared with untreated control mice, aged nmEV‐treated mice exhibited a significantly improved trabecular architecture, including increases in BV/TV, Tb.N, and BMD. These findings collectively highlight the broad spectrum and rhythm‐synchronizing effects of nmEVs as a promising intervention for age‐associated functional decline.

While our findings strongly support the role of nmEVs in reestablishing circadian rhythm at the molecular level, as evidenced by the restored expression of core clock proteins such as Bma1 and Clock, our data also partially elucidate the critical role of the p53 signaling pathway. Specifically, we found that the modulation of p53 activity influenced the ability of nmEVs to restore rhythmic gene expression, highlighting p53 as a potential mediator connecting cellular senescence to circadian disruption. We further extended these observations to the behavioral level through voluntary wheel running analysis. Treated mice not only exhibited improved physical performance but also displayed realigned rest‒activity cycles, with increased activity during the light phase and reduced activity during the dark phase, suggesting a partial restoration of circadian behavior. Nevertheless, a limitation in our current study was that we did not directly assess sleep‐wake states using neurophysiological tools such as EEG. Given the pivotal role of the brain's circadian clock in regulating sleep and global homeostasis, future investigations will incorporate 24‐h video tracking, infrared‐based motion monitoring, and EEG‐based assessments to more precisely characterize how nmEVs impact sleep architecture, circadian phase coherence, and central rhythm entrainment. This will provide a more comprehensive and mechanistic understanding of how molecular reprogramming translates into systemic and neurobehavioral rhythmicity during aging.

Despite the growing enthusiasm for EV‐based antiaging therapies, their clinical translation remains constrained by practical limitations, particularly in elderly populations where autologous cell sourcing is inherently challenging.^[^ ^35^ ^]^ In aged individuals, the procurement and expansion of high‐quality stem cells or progenitor cells are often hindered by donor frailty, reduced cell proliferation, and senescence‐associated phenotypes. These limitations significantly impair EV yield and reproducibility, making it difficult to generate sufficient quantities for therapeutic use. Furthermore, while allogeneic or xenogeneic EVs offer a potential workaround, concerns surrounding long‐term immunogenicity, donor heterogeneity, and ethical sourcing remain unresolved.^[^ ^36^ ^]^ These barriers highlight the pressing need for an alternative EV source that is ethically accessible, minimally invasive, and independent of donor age.

In contrast, our study leveraged the nasal mucosa as a unique EV source on the basis of clinical observations that this tissue exhibits remarkable regenerative capacity independent of chronological age. Unlike traditional stem cell sources, the nasal mucosa can be safely, ethically, and repeatedly sampled, making it a highly accessible and sustainable EV reservoir. While nasal mucosa‐derived EVs may carry mixed‐lineage signatures, their nonage‐dependent regenerative profile and immunological compatibility substantially increase their translational potential for broad‐spectrum antiaging applications. Nevertheless, future clinical translation will require careful ethical oversight of human tissue procurement and rigorous safety evaluation of allogeneic EV administration, particularly with regard to immunogenicity and long‐term tolerability.

Despite these advantages, several important questions remain to be addressed. As the nasal mucosa is a complex tissue composed of multiple cell types, nmEVs likely represent a heterogeneous vesicle population, and it remains unclear which specific cell‐derived EV subtypes, such as epithelial, basal stem, or immune‐associated cells, play dominant roles in mediating the observed antiaging effects. Future studies involving single‐cell lineage tracing and EV subfractionation will be crucial to deconvolute this heterogeneity. Furthermore, although we focused on organ‐level and systemic phenotypes, the immunomodulatory role of nmEVs was not thoroughly explored in this study and warrants detailed investigation, particularly given the central involvement of inflammation in aging. Finally, whether stem cells and enriched subpopulations within the nasal mucosa also release EVs with intrinsic antiaging effects, which may represent a valuable and more defined therapeutic subset for future applications, remains to be determined. In addition to the issue of cellular origin, a central question is which bioactive components within nmEVs are responsible for their anti‐aging effects. At present, these molecular effectors remain undefined. Small non‐coding RNAs, particularly microRNAs, are likely candidates given their ability to modulate p53 signaling and other senescence‐associated pathways. Specific proteins involved in stress responses, chromatin regulation, or metabolic control may also contribute to the observed systemic benefits. Future studies combining proteomic and transcriptomic profiling with functional validation will be required to identify the critical cargo mediating these effects.

## 4 Conclusion

In summary, this study provided compelling evidence that nmEVs exert systemic antiaging effects across multiple levels, from behavior and organ structure to molecular and cellular hallmarks of aging. Through mechanisms involving the suppression of p53 signaling and the reestablishment of circadian regulation, the nmEVs improved tissue homeostasis and regenerative capacity in aged mice. Their accessibility, safety, and multilineage regenerative profile offer significant advantages over those of traditional EV sources. These findings not only establish the regenerative potential of nmEVs but also provide a conceptual and technological foundation for advancing tissue‐derived EVs into next‐generation, broad‐spectrum antiaging therapies with high clinical feasibility.

## 5 Experimental Section

### Statistical Analysis

All data are presented as mean ± standard deviation (SD) unless otherwise specified. Before hypothesis testing, data distributions were checked (Shapiro‐Wilk) and variances assessed (Levene); when assumptions were violated, values were log10‐transformed (or arcsine–square‐root transformed for proportions) and, if still non‐normal, analyzed with nonparametric tests as indicated. Outliers were prespecified and screened using the ROUT method (Q = 1%) in GraphPad Prism with decisions made blinded to group labels. Unless noted, tests were two‐sided with α = 0.05. Comparisons between two groups used two‐tailed unpaired Student's *t*‐tests. Comparisons among more than two groups (e.g., Young, Aged, nmEVs) used one‐way ANOVA with Tukey's post hoc test (or Kruskal–Wallis with Dunn's test for nonparametric data). Time‐course behavioral data (e.g., Morris Water Maze learning phase) were analyzed by two‐way repeated‐measures ANOVA with Bonferroni correction; sphericity was checked by Mauchly's test and Greenhouse‐Geisser correction applied when violated. Paired analyses of hBMSCs from the same donor before and after nmEVs treatment used two‐tailed paired *t*‐tests (or Wilcoxon signed‐rank tests if nonparametric). For transcriptomic datasets, differential expression was computed in DESeq2 with the median‐of‐ratios normalization and Wald tests; multiple testing in gene‐ and pathway/GO‐level analyses was controlled by the Benjamini–Hochberg procedure, with adjusted P < 0.05 considered significant. The biological replicate number (n) for each analysis is reported in the corresponding figure legends; technical replicates were averaged to yield one value per biological replicate. All statistical analyses were conducted in GraphPad Prism (version 10.0).

### Ethics Approval Statement

All experimental procedures involving human and animal subjects were conducted in strict accordance with ethical guidelines and were approved by the relevant institutional review boards.

Human nasal mucosal tissues were obtained from adult donors with written informed consent in compliance with the Declaration of Helsinki and with approval from the Ethics Committee of Jiangnan University Medical Center, Wuxi No. 2 People's Hospital Ethics Committee (approval no. Y‐108).

Human bone marrow‐derived mesenchymal stem cells were isolated from the femoral heads of elderly patients who underwent hip arthroplasty, with informed consent and approval from the Ethics Committee of Jiangsu University Affiliated Gaochun Hospital (approval no. 2024‐129‐01). All participants were fully informed of the study's purpose and procedures, and their identities and privacy were strictly protected

All animal experiments were conducted in accordance with the institutional guidelines for the care and use of laboratory animals and were approved by the Animal Ethics Committee of Jiangnan University (JN.No20241230c0720715[703]). The animals were housed under standard conditions, and all efforts were made to minimize animal suffering and reduce the number of animals used.

### Patient Consent Statement

Informed consent was obtained from all individual participants included in the study.

The detailed materials and methods are provided in Supporting Information 2.

## Conflict of Interest

The authors declare no conflict of interest.

## Supporting information



Supporting Information

## Data Availability

The data that support the findings of this study are available from the corresponding author upon reasonable request.
