# Supplementary material for: Nasal Mucosa‐Derived Extracellular Vesicles as a Systemic Antiaging Intervention
Source: Adv Sci (Weinh). 2025 Nov 21;13(5):e11372. doi: 10.1002/advs.202511372 (PMC12850021; doi:10.1002/advs.202511372)
Supplement: Supplementary file 1 — Supporting Information [file ADVS-13-e11372-s001.docx]

**Human Nasal Mucosa Sampling and Processing**

Human nasal mucosa samples were endoscopically harvested from the respiratory region of the nasal septum adjacent to the inferior turbinate. The procedure was approved by the Ethics Committee of Jiangnan University Affiliated Hospital (Wuxi No.2 People’s Hospital) (approval ID: Y-108), and written informed consent was obtained from all participants. To preserve the native microenvironment and physiological characteristics of the tissue, no enzymatic digestion was applied. Instead, the mucosal tissues were finely minced into approximately 1 mm^3^ fragments and directly placed into serum-free DMEM/F12 medium.

**EV Isolation from Nasal Mucosa Medium**

Medium from nasal mucosa explants was collected every 48 h and kept at 4 °C (processed within 24 h). The medium was clarified by sequential centrifugation at 4 °C: 300 × g for 10 min (remove cells), 2,000 × g for 20 min (cell debris), and 10,000 × g for 30 min (large vesicles/organelles). The supernatant was optionally passed through a 0.22 µm PES filter (low protein-binding). The pre-cleared medium was ultracentrifuged at 100,000 × g for 70 min at 4 °C in polyallomer tubes (Beckman Coulter SW41 Ti; record rotor and k-factor). The supernatant was discarded, and the pellet was gently resuspended in cold PBS (0.5–1.0 mL), transferred to a clean tube, and washed by a second spin at 100,000 × g for 70 min at 4 °C. The final EV pellet was resuspended in PBS (typically 50–200 µL per T-175 equivalent), aliquoted, and stored at −80 °C (≤2 freeze–thaw cycles). For reporting, particle number (by NTA) and protein mass (by BCA) were recorded; particle-to-protein ratios were used as a purity check.

**Nanoparticle Tracking Analysis**

EV samples were thawed on ice, gently mixed, and clarified at 2,000 × g for 10 min at 4 °C to remove storage-induced aggregates. Aliquots were diluted in 0.22 µm-filtered PBS to yield ~50–80 particles per frame during acquisition. Measurements were performed at room temperature (22–25 °C) on a NanoSight NS300 (Malvern Instruments Ltd., UK). For each sample, camera level and detection threshold were kept constant across runs (e.g., 12–14 and 5–7, respectively), and three 60-s videos were recorded in static mode (flow = 0) at 25 fps. Videos were analyzed in batch with identical settings; particle size (mode, D10/D50/D90) and concentration (particles/mL) were reported as mean ± SD after multiplying by the dilution factor. The chamber was flushed with filtered PBS (≥3 volumes) between samples. If particle density exceeded 80/frame or fell below 30/frame, the dilution was adjusted and the sample re-measured; acquisitions showing drift or bubbles were discarded and repeated.

**Transmission Electron Microscopy**

EV suspensions (~1–5 × 10^9 particles/mL) were adsorbed onto glow-discharged Formvar/carbon-coated 200-mesh copper grids by placing 10 µL on the grid for 1–2 min. Excess liquid was wicked off with filter paper. Grids were fixed with 2% paraformaldehyde in PBS for 10 min, washed twice with PBS, and negatively stained with 2% uranyl acetate for 60 s (alternative: 1% phosphotungstic acid, pH 7.0, 60–90 s). After air-drying, grids were imaged on a transmission electron microscope operated at 100 kV (Hitachi HT7800). For each preparation, at least five fields from two independent grids were captured; representative vesicles with intact, cup-shaped morphology and diameters in the expected range were documented.

**Western Blotting for EV Markers**

EVs were lysed in ice-cold RIPA buffer (50 mM Tris-HCl pH 7.4, 150 mM NaCl, 1% NP-40, 0.5% sodium deoxycholate, 0.1% SDS) supplemented with protease/phosphatase inhibitors. Lysates were clarified at 12,000 × g for 10 min at 4 °C, and protein content was determined by BCA assay. Samples were mixed 1:3 with 4× Laemmli buffer containing 100 mM DTT (or 5% β-mercaptoethanol) and heated at 70 °C for 10 min. Equal protein (15 µg per lane) was resolved on 10–12% SDS-PAGE and transferred to PVDF membranes (0.45 µm) using wet transfer at 100 V for 60 min on ice. Membranes were blocked in 5% non-fat milk in TBST (20 mM Tris-HCl pH 7.6, 150 mM NaCl, 0.1% Tween-20) for 1 h at room temperature, then incubated overnight at 4 °C with primary antibodies against EV-positive markers, including CD63 (Cell Signaling Technology, #52090, 1:1000) and CD9 (Cell Signaling Technology, #13174, 1:1000). After TBST washes (3 × 10 min), membranes were incubated with HRP-conjugated secondary antibodies (boster; BA1055; 1:5000) for 1 h at room temperature, washed, and developed with ECL reagent. Chemiluminescence was captured on a digital imager with exposure ranges noted.

**Animal studies and experimental design**

Female C57BL/6J mice were randomly allocated into three groups: (i) young control group (10 months at baseline, sacrificed at 12 months); (ii) aged control group (20 months at baseline, sacrificed at 22 months); and (iii) nmEVs treatment group (20 months at baseline, sacrificed at 22 months). Mice in the treatment group received tail vein injections of 30 μg nmEVs (corresponding to approximately 2-3 × 10^9^ particles, as determined by NTA) diluted in 100 μL PBS, administered twice weekly for 8 consecutive weeks. Both the aged control group and the young control group were injected with equal volumes of PBS on the same schedule. All animal procedures were reviewed and approved by the Institutional Animal Care and Use Committee of Jiangnan University (IACUC approval ID: JN.No20241230c0720715[703]) and were performed in accordance with institutional guidelines.

**Morris water maze test**

Spatial learning and memory performance were assessed using the Morris water maze. Mice underwent five days of acquisition training with four trials per day, each starting from a pseudo-randomized location. Each trial lasted a maximum of 60 seconds, and the latency to locate the submerged platform was recorded. Mice failing to locate the platform within 60 seconds were guided to it and allowed to stay for 15 seconds. On the sixth day, the platform was removed for the probe test. The time spent in the target quadrant, number of platform crossings, and swimming path were recorded using an automated video tracking system. All tests were conducted under consistent lighting and temperature conditions.

**Voluntary wheel running and treadmill endurance test**

Circadian locomotor activity was measured using voluntary wheel running. Mice were housed individually in cages equipped with low-resistance running wheels, and wheel revolutions were continuously recorded for 7 days. Hourly running distance data were binned and analyzed based on light/dark phase (ZT0-12 vs. ZT12-24). Treadmill endurance was evaluated using an automated treadmill device. After acclimation, mice were subjected to a fixed speed endurance test until exhaustion, defined as remaining on the shock grid for more than 5 seconds despite mechanical encouragement.

**Assessment of neuromuscular strength and motor coordination**

Neuromuscular strength was measured using a digital grip strength meter (Panlab, Harvard Apparatus), which detects the maximum force exerted by mice when pulling against a horizontal metal bar. Each mouse was allowed to grasp the bar with its forepaws, and was then gently pulled backward until its grip was released. The device automatically recorded the peak tension in gram-force units. Three consecutive trials were conducted for each animal, and the average value was used for statistical analysis.

Motor coordination was evaluated using a Rota Rod system (Panlab, Harvard Apparatus, model no. 76-0772). Mice were placed on a rotating rod with a fixed width of 3 cm that accelerated gradually from low to high speed. The time until the animal fell from the rod was recorded in seconds. Each mouse was tested in three independent sessions with sufficient rest between trials, and the average latency to fall was calculated as the final performance score.

**Cellular assays in hBMSCs**

Human bone marrow-derived mesenchymal stem cells (hBMSCs) were isolated from femoral heads of healthy elderly donors (age >65) undergoing hip replacement surgery due to femoral neck fracture. All procedures were approved by the Ethics Committee of Gaochun Hospital, Jiangsu University (approval ID: 2024-129-01), and written informed consent was obtained from all participants prior to sample collection. Bone marrow was flushed from the femoral head, filtered, and cultured using the standard plastic adherence method in DMEM supplemented with 10% FBS. After reaching confluence, cells were passaged and characterized by immunofluorescence staining for MSC markers (CD44, CD105) and negative markers (CD14,). Passage 5 cells (P5) exhibited reduced proliferation and increased Senescence-associated β-galactosidase (SA-β-Gal) positivity and were used as the senescence model. nmEVs were added at a concentration of 10 μg/mL for 48 hours. Senescence markers (p21, p21, and p53), SASP components (IL-1) were assessed by Western blot and immunofluorescence.

**Senescence-associated β-galactosidase staining**

SA-β-Gal staining was performed using a commercial kit (Beyotime) following the manufacturer's protocol. hBMSCs were fixed in fixative solution for 15 minutes, incubated with X-gal staining solution at 37 °C (without CO_2_) overnight, and observed under a bright-field microscope. Blue-stained cells were counted as β-Gal-positive senescent cells.

**Immunofluorescence staining**

Cells were seeded on glass coverslips and fixed in 4% paraformaldehyde for 15 minutes. After permeabilization with 0.2% Triton X-100, cells were blocked in 3% BSA and incubated with primary antibodies overnight at 4 °C. After washing, cells were incubated with fluorescently labeled secondary antibodies for 1 hour at room temperature, counterstained with DAPI, and mounted using antifade medium. Images were captured using a confocal laser scanning microscope, and positive cell percentages were quantified in ImageJ.

**Osteogenic differentiation of hBMSCs**

To evaluate osteogenic potential, hBMSCs were subjected to osteogenic induction for 21 days with or without nmEVs treatment. Early and late-stage osteogenesis were assessed using alkaline phosphatase (ALP) and Alizarin Red S (ARS) staining, respectively. Protein expression of osteogenic markers Opn and Osx was confirmed by Western blot.

**Histology and immunohistochemistry**

Tissue samples from the brain, liver, lung, kidney, and bone were fixed in 4% paraformaldehyde, embedded in paraffin, and sectioned. Standard hematoxylin and eosin (H&E) and Masson’s trichrome were performed to assess tissue morphology and fibrosis. Immunohistochemistry was conducted using antibodies against p21, il-1, and pai-1. Sections were developed with DAB and counterstained with hematoxylin. Quantification was performed using ImageJ.

**Western blotting**

Proteins were extracted from tissues or cells using RIPA buffer, resolved by SDS-PAGE, and transferred to PVDF membranes. Membranes were incubated with primary antibodies against p21, p53, Bmal1, Clock, Opn, and Osx, followed by HRP-conjugated secondary antibodies. Bands were visualized using chemiluminescence and quantified by densitometry.

**Single-nucleus RNA sequencing and analysis**

Hippocampal tissues were dissected and immediately placed in ice-cold PBS. After removing blood vessels and meninges, the tissues were minced into ~1 mm^3^ pieces and enzymatically digested in a solution containing 1 mg/mL collagenase I, 1 mg/mL collagenase II, 60 U/mL hyaluronidase, 10 U/mL liberase, and 0.02 mg/mL DNase I at 37 °C for 90 minutes with gentle agitation. The resulting suspension was filtered sequentially through 100 μm and 40 μm cell strainers. Red blood cells were lysed using RBC lysis buffer, and the remaining cells were washed with DPBS containing 0.5% BSA.

Nuclei were isolated using a mild detergent-based lysis buffer, followed by centrifugation and filtration. The isolated nuclei were counted and viability assessed under a microscope with trypan blue staining. Approximately 8,000–10,000 nuclei per sample were loaded onto a Chromium Next GEM Chip G for single-nucleus partitioning and barcoding using the Chromium Single Cell 3' Reagent Kit v3.1 (10x Genomics). Reverse transcription and cDNA amplification were performed according to the manufacturer’s protocol. Libraries were constructed and sequenced on an Illumina NovaSeq 6000 platform.

Raw sequencing data were processed using the Cell Ranger pipeline (10x Genomics, v6.0) for demultiplexing, alignment to the mouse reference genome (mm10), and generation of feature-barcode matrices. Further downstream analysis was conducted using Scanpy (v1.8) in Python. Low-quality nuclei with fewer than 100,000 UMIs or with >25% mitochondrial gene content were filtered out. Normalization was performed using pp.normalize_total, followed by logarithmic transformation using pp.log1p. Highly variable genes were identified with the Macosko method, and batch correction was performed if necessary. Principal component analysis (PCA) was used for dimensionality reduction, and neighborhood graph construction was achieved with pp.neighbors. Clustering was performed using the Louvain algorithm, and UMAP was used for visualization.

Differentially expressed genes across clusters were identified using the Wilcoxon rank-sum test in Seurat (Find AllMarkers function), with thresholds set at adjusted p < 0.05 and |log₂FC| > 0.25. Cell type annotation was based on canonical marker genes and reference atlas comparison. Functional enrichment analysis, including Gene Ontology (GO) and Kyoto Encyclopedia of Genes and Genomes (KEGG), was performed using the R package ClusterProfiler.

**RNA sequencing of major organs**

Total RNA was extracted from the heart, liver, spleen, lung, and kidney tissues using TRIzol reagent (Invitrogen, USA) according to the manufacturer’s protocol. RNA purity and concentration were assessed using a Nanodrop spectrophotometer and Agilent Bioanalyzer 2100. Libraries were prepared using a standard poly(A) enrichment protocol, followed by reverse transcription and second-strand synthesis. Sequencing was performed on the Illumina NovaSeq 6000 platform with paired-end 150 bp reads.

Raw reads were subjected to quality control and aligned to the mouse reference genome (mm10) using HISAT2. Gene expression levels were quantified using featureCounts, and differential expression analysis was conducted using DESeq2. Genes with adjusted p-values < 0.05 and |log₂FC| > 1 were considered differentially expressed. Functional enrichment analyses, including GO and KEGG pathway analysis, were performed using the ClusterProfiler R package.

**Agents table**

| REAGENT or RESOURCE | SOURCE | IDENTIFIER |
| --- | --- | --- |
| Antibodies | | |
| CD44 | Santa cruz | sc-7297 |
| CD14 | Santa cruz | sc-19588 |
| CD133 | Santa cruz | sc-376764 |
| Opn | Proteintech | 22952-1-AP |
| p16 | CST | 18769 |
| p21 | CST | 2947 |
| p53 | CST | 9282 |
| Bmal1 | CST | 14020 |
| Osx | Proteintech | 28694-1-AP |
| clock | Proteintech | 82829-1-RR |
| anti-mouse-CY3 | Boster | [BA1031](https://www.boster.com.cn/home/product/cy3-conjugated-affinipure-goat-anti-mouse-igg-h-l_ba1031.html) |
| anti-rabbit-488 | Boster | [BA1127](https://www.boster.com.cn/home/product/dylight-488-conjugated-affinipure-goat-anti-rabbit-igg-h-l_ba1127.html) |
| anti-rabbit-CY3 | Boster | [BA1032](https://www.boster.com.cn/home/product/cy3-conjugated-affinipure-goat-anti-rabbit-igg-h-l_ba1032.html) |
| anti-mouse-488 | Boster | [BA1126](https://www.boster.com.cn/home/product/dylight-488-conjugated-affinipure-goat-anti-mouse-igg-h-l_ba1126.html) |
|  |  |  |
| Reagents |  |  |
| Alkaline Phosphatase Kit | biyuntian | P0321S |
| Alizarin red S | biyuntian | C0140M |
| Cck-8 | biyuntian | C0037 |
| β-gal kit | biyuntian | C0602 |
| DMEM/F12 | Cytiva HyClone | SH30023 |
| L-ascorbic acid 2-phosphate | MCE | [HY-103701](https://www.medchemexpress.cn/l-ascorbic-acid-2-phosphate.html) |
| dexamethasone | MCE | HY-14648 |
| β-glycerophosphate disodium | MCE | HY-126304 |
| fetal bovine serum | BIOEXPLORER®Life Sciences | BS1618-10 |
| Nutlin-3a | MCE | HY-10029 |
| Ultrapure RNA Kit | YEASEN | 19231ES50 |
|  |  |  |
|  |  |  |
